# Supplementary material for: State-selective small molecule degraders that preferentially remove aggregates and oligomers
Source: Nat Commun. 2025 Nov 25;16:10486. doi: 10.1038/s41467-025-65454-z (PMC12647892; doi:10.1038/s41467-025-65454-z)
Supplement: Supplementary file 1 — Supplementary Information [file 41467_2025_65454_MOESM1_ESM.pdf]

State-selective small molecule degraders that preferentially remove aggregates and oligomers

Supplementary Information File

Supplementary Figures

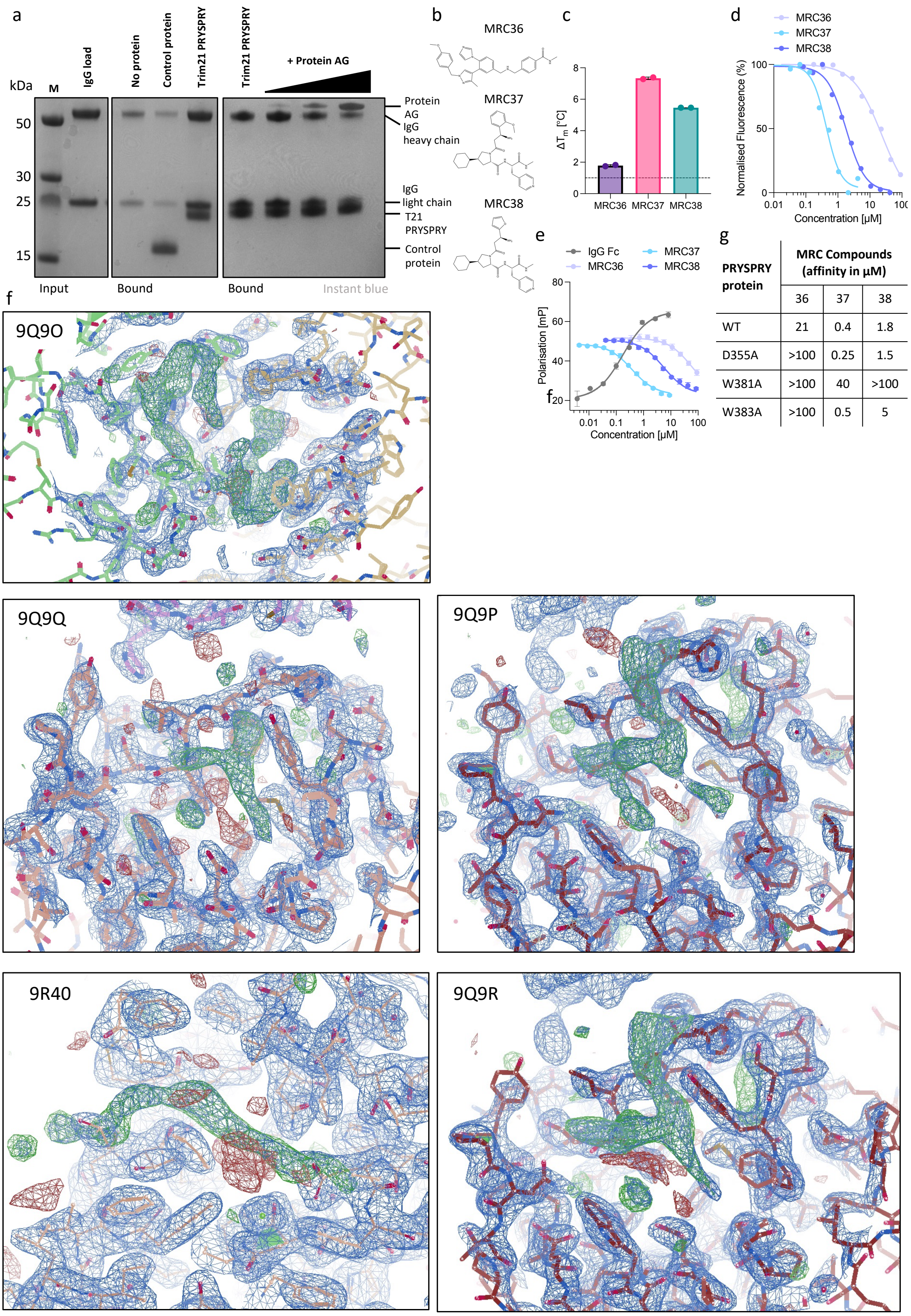

PRYSPRY protein	MRC Compounds (affinity in µM)		
	36	37	38
WT	21	0.4	1.8
D355A	>100	0.25	1.5
W381A	>100	40	>100
W383A	>100	0.5	5

Supplementary Figure 1: Identification of ligands for TRIM21 PRYSPRY. (a) SDS-PAGE gels stained with Instant blue showing that the activity of T21 PRYSPRY is maintained when covalently bound to beads; left: shows the amount of IgG that can be recovered in the experiment. Middle: Beads reacted with either no protein, control protein or T21 PRYSPRY was incubated with the IgG, washed and the bound fraction analysed. Right: Similar as the middle experiment but where Protein AG was included when beads were incubated with IgG to show that binding to TRIM21 PRYSPRY is specific. **(b)** MRC37, MRC37 and MRC38 compound structures. **(c)** Thermal stabilisation of TRIM21 PRYSPRY by indicated ligands as compared to melting temperature in the presence of solvent (DMSO) alone. Plotted difference in melting temperature (ΔT_m) as an average (± s.e.m.) of two independent replicates (n=2). **(d)** Tryptophan quenching experiment, where the compounds are titrated against a constant T21 PRYSPRY and the intrinsic fluorescence of the protein is monitored to follow binding of the compounds to the protein. Data is expressed as a percentage of fluorescence (RFU) in the absence of compound. **(e)** Fluorescence polarization displacement experiment, where T21 PRYSPRY labelled with Alexa-488 is bound to IgG Fc and titrated against the compounds to determine if they can displace Fc, as determined by the change in polarisation of the fluorescence. The full black circles show a titration of IgG Fc against 488-labelled T21 PRYSPRY. Representative experiment of at least two independent experiments (n=2) **(f)** Omit map (FOFC contoured at 3 sigma, green) and example electron density map (2FOFC contoured at 1.2 sigma, blue) for indicated pdb. **(g)** Affinity of indicated compounds to wild-type (WT) T21 PRYSPRY and selected mutants as measured by quenching of intrinsic tryptophan fluorescence. Values are Kd in µM.

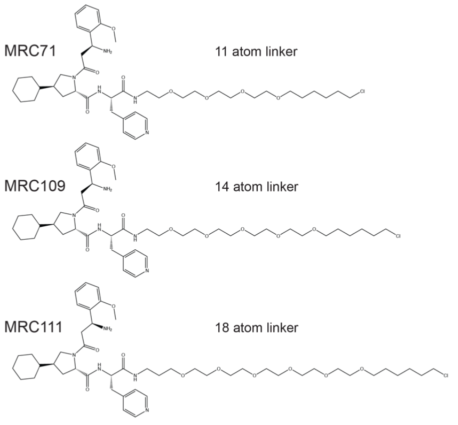

Supplementary Figure 2: haloTRIMTAC compounds. Compound structures for MRC71, MRC109 and MRC111.

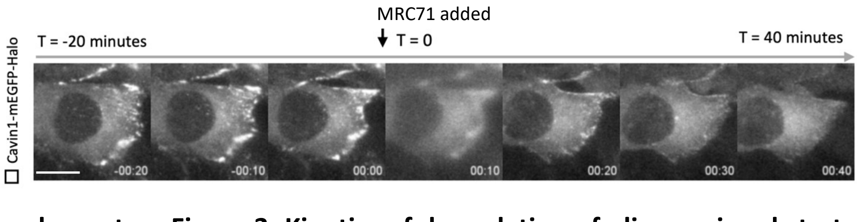

Supplementary Figure 3: Kinetics of degradation of oligomeric substrate Cavin1-mEGFP-Halo. RPE1 cells expressing Cavin1-mEGFP-Halo were treated with haloTRIMTAC MRC71 and the fluorescence monitored using live microscopy. An exemplar cell showing that the oligomeric membrane associated protein is rapidly degraded but diffuse cytoplasmic protein remains. Scale bar = 10 µm.

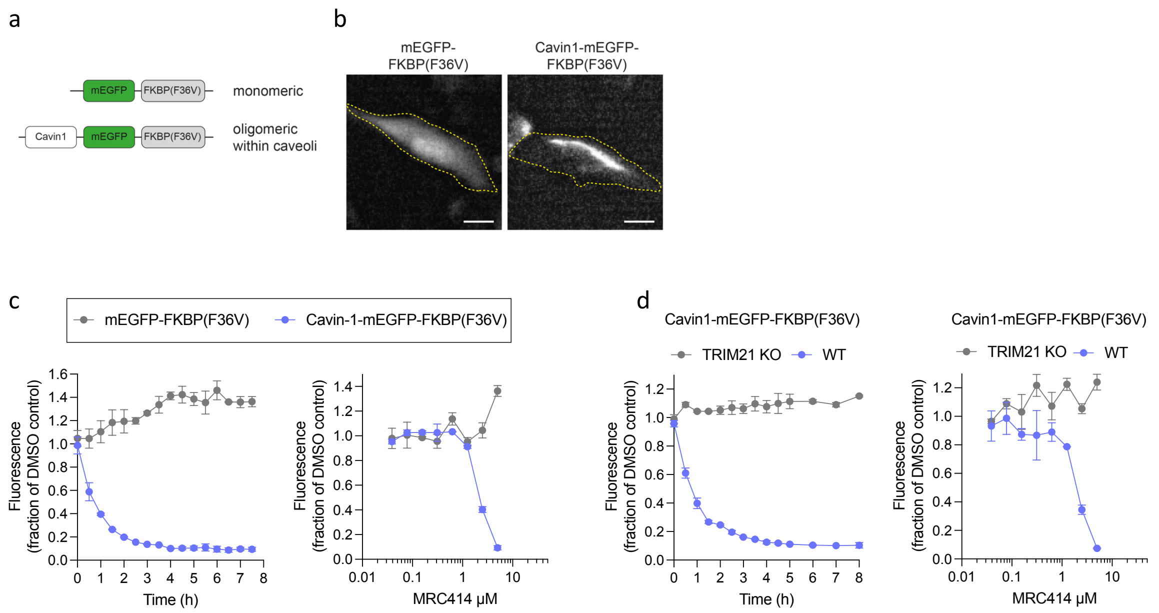

Supplementary Figure 4: dTAG-TRIMTAC rapidly and efficiently degrades Cavin1-mEGFP-FKBP(F36V). (a) Two different FKBP(F36V)-tagged targets for TRIMTACs; mEGFP-FKBP(F36V) is a monomeric protein, Cavin1-mEGFP-FKBP(F36V) forms oligomers within caveoli membrane structures. **(b)** Representative images of RPE-1 stable cell lines expressing the difference protein constructs; mEGFP-FKBP(F36V) exhibits diffuse cytosolic localisation, Cavin1-mEGFP-FKBP(F36V) exhibits bright signal at the cell membrane indicative of caveoli structures. Yellow dotted line shows the cell outline. **(c-d)** MRC414 compounds selectively degrade oligomeric Cavin1-mEGFP-FKBP(F36V) over monomeric mEGFP-FKBP(F36V) and degradation is TRIM21 dependent (d). Graphs show the integrated density of GFP fluorescence (in relative fluorescence units, RFU) normalized to total cell area (phase) from 1 x 10^4 cells and expressed as a fraction of the DMSO control. Data is expressed as mean and s.e.m. from *n* = 4 technical replicates. Representative examples from *n* = 2 independent experiments.

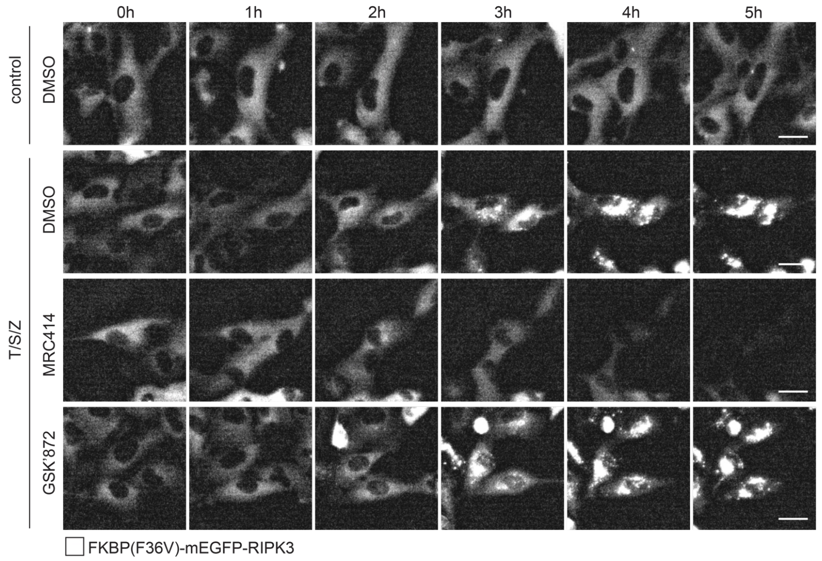

Supplementary Figure 5: dTAG-TRIMTAC degrades RIPK3 oligomers as they assemble. Live imaging of mEGFP-FKBP(F36V)-RIPK3 shows that MRC414 degrades RIPK3 oligomers as they assemble whereas GSK'872 does not affect RIPK3 oligomerization. Scale bar = 20 µm.

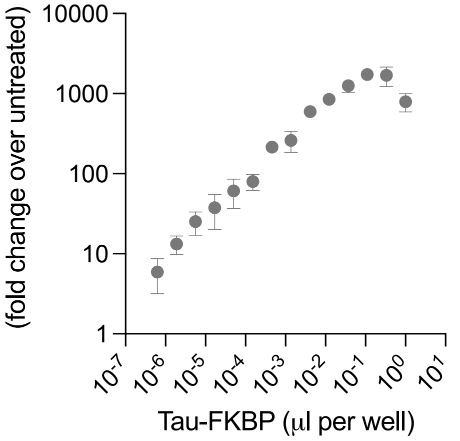

Supplementary Figure 6: FKBP(F36V)-tau assemblies induce accumulation of intracellular pS422-positive aggregates. Neural cultures were treated with indicated volumes of FKBP(F36V)-tau assemblies for 7 days, followed by methanol fixation and staining with the MAP2 and pS422 (anti-

	**9Q9O**: TRIM21:MRC36	**9Q9P**: TRIM21:MRC37	**9Q9Q**: TRIM21:MRC38	**9Q9R**: TRIM21:MRC209	**9R40** HaloTag:MRC71
**Data collection**					
Space group	P1211	P1211	P1211	P1211	P43212
Cell dimensions					
a, *b*, *c* (Å)	60.7, 74.4, 90.1	60.6, 41.9, 90.0	45.3, 60.6, 66.7	37.1, 60.8, 42.2	62.8, 62.8, 163.7
α, β, γ (°)	90, 90.1, 90	90, 113.0, 90.0	90.0, 99.2, 90.0	90.0, 112.5, 90.0	90.0, 90.0, 90.0
Resolution (Å)	50.43-2.46 (2.46-2.56)	38.59-2.10 (2.16-2.10)	44.64-2.12 (2.15-2.12)	30.41 - 2.33 (2.37-2.33)	58.61-2.04 (2.08-2.04)
R_{meas}	N/A	N/A	N/A	N/A	N/A
$CC_{1/2}$ (%)	100 (20)	96 (30)	100 (10)	90 (20)	100(10)
$I / \sigma I$	3 (0.3)	4.8 (1.1)	3.6 (0.1)	5.4 (0.8)	4.8 (0.2)
Completeness (%)	83.6 (36)	100 (100)	88.9 (50.3)	99.7 (93.7)	100 (94.9)
Redundancy	3.1 (2.2)	9.0 (9.9)	2.8 (1.8)	3.5 (3.6)	10.1 (3.1)
**Refinement**					
Resolution (Å)	2.46	2.1	2.12	2.33	2.04
No. reflections	24473	10082	18182	7432	21548
R_{work} / R_{free}	0.31/0.38	0.23/0.30	0.30/0.35	0.22/0.32	0.25/0.30
No. atoms	6096	1555	3040	1530	
Protein	5940	1485	2962	1475	2359
Ligand/ion	156	39	72	44	56/1
Water	0	31	6	11	35
B-factors					
Protein	36.01	35.38	28.03	38.21	38.4
Ligand/ion	27.54	25.53	21.66	40.52	121.14/32.72
Water	N/A	31.71	11.4	21.98	35.09
R.m.s. deviations					
Bond lengths (Å)	0.01	0.01	0.01	0.01	0.01
Bond angles (°)	1.7	1.49	1.49	1.58	1.56

*Values in parentheses are for highest-resolution shell.

Supplementary Table 1: Data collection and refinement statistics (molecular replacement). Full statistics are given for each of the complexed structures, together with the PDB codes.

Supplementary Methods

Chemistry Supporting Information

I. GENERAL EXPERIMENTAL DETAILS

All reagents were purchased from commercial suppliers and used without further purification unless otherwise stated. Reactions requiring air-sensitive reagents and dry solvents were performed in glassware that had been dried in an oven at 150 °C prior to use. These reactions were carried out under argon or nitrogen atmosphere with the exclusion of air. Reactions were monitored by thin-layer chromatography (TLC) on Merck silica gel 60 covered aluminium sheets. TLC plates were visualised under UV-light, and where required with an acidic ethanolic anisaldehyde solution, acidic butanolic ninhydrin solution, a $KMnO_4$ solution or a bromo-cresol green solution. NMR spectra were recorded on a Bruker DPX-400 spectrometer (^{1}H NMR at 400 MHz or ^{13}C NMR at 101 MHz). Chemical shifts are reported in ppm. ^{1}H NMR spectra were recorded with chloroform-*d*, methanol-d_4 or DMSO-d_6 as the solvent using residual $CHCl_3$ (δ = 7.26), or CHD_2OD (δ = 3.31) or $(CHD_2)SOCD_3$ (δ = 2.50) as internal standard, and for ^{13}C NMR spectra the chemical shifts are reported relative to the central resonance of $CDCl_3$ (δ = 77.16), CD_3OD (δ = 49.00) or $(CD_3)_2SO$ (δ = 39.52). Signals in the obtained spectra are reported as singlet (s), doublet (d), triplet (t), quartet (q), quintet (p), septet (hept), multiplet (m), broad (br), or a combination of these, to describe the observed spin–spin coupling pattern. Spin–spin coupling constants are reported in Hertz (Hz) and are uncorrected. Two-dimensional NMR spectroscopy (COSY, HSQC or HMBC) was employed where appropriate to assist the assignment of signals in the ^{1}H and ^{13}C NMR spectra. The selected assigned resonances were used to confirm connectivity or transformation. Semi-preparative reverse-phase HPLC was performed on a Gilson HPLC system equipped with Gilson 306 pumps, a Phenomenex Synergi C18 (80 Å, 10 µm, 250×21.2 mm) column at a flow rate of 10 mL min^{-1}. Non-linear gradients between 50% to 95% HPLC grade acetonitrile in ultra-pure water with 0.1% formic acid were utilised. UV absorption was detected at 214 nm and 254 nm using a Gilson 155 UV/VIS detector. Collected fractions were then lyophilised using a Christ Alpha 2-4 LDplus lyophiliser. IR spectra were obtained on a JASCO FTIR-4100 instrument with a Golden Gate™ attachment using a type IIa diamond as a single reflection element for the IR spectra of the solid or liquid compounds to be detected directly (thin layer). High-resolution mass spectra (HRMS) were recorded using ESI conditions by the analytical services at the University of Glasgow. Liquid chromatography–mass spectrometry (LCMS) was recorded on an Agilent 6125B Single Quad LC-MS mass spectrometer, coupled with an Agilent 1290 Infinity UHPLC chromatography system using positive mode electrospray ionisation (ESI+) or negative mode electrospray ionisation (ESI-). A Dr Maisch GmbH Reprosil Gold 120 C18 (3 µm 150×4 mm) column was used with UV absorption detected

at 214 nm and 254 nm. Linear gradients between 5% to 90% HPLC-grade acetonitrile in ultra-pure water with 0.1% formic acid over 15 minutes were utilised with a flow rate of 0.3 mL min^{-1}. Final compound purity was assessed by LCMS as >95%.

II. PREPARATION OF MRC37

Ethyl (2*S*,4*S*)-cyclohexylpyrrolidine-2-carboxylate (1)

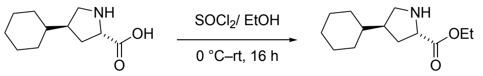

1

To a stirred solution of *trans*-4-cyclohexyl-L-proline (100 mg, 0.507 mmol, 1.00 equiv.) in ethanol (2.5 mL, 0.20 M) at 0 °C was added thionyl chloride (0.111 mL, 1.52 mmol, 3.00 equiv.) dropwise. The resulting reaction mixture was left to stir at room temperature for 18 h. The reaction mixture was concentrated *in vacuo* (toxic Buchi) to give desired ester **1** as a white solid (130 mg, 98%).^{1}H NMR (400 MHz, $CDCl_3$) δ_H: 0.84–1.38 (9H, m), 1.56–1.78 (5H, m), 1.94–2.10 (2H, m), 2.24–2.38 (1H, m), 3.06 (1H, br s), 3.75 (1H, br s), 4.28 (2H, q, *J* 7.1 Hz), 4.48 (1H, br s), 8.88 (1H, br s), 11.11 (1H, br s); ^{13}C{^{1}H} NMR (101 MHz, $CDCl_3$) δ_C: 14.2 (CH_3), 25.9 (2 × CH_2), 26.2 (CH_2), 31.6 (CH_2), 32.0 (CH_2), 33.3 (CH_2), 41.1 (CH_3), 43.1 (CH_3), 49.4 (CH_2), 59.4 (CH_3), 63.2 (CH_2), 169.1 (C); HRMS (ESI) $C_{13}H_{24}NO_2^+$ ([M+H$^+$]) requires 226.1802, found 226.1806 (1.7 ppm)

Methyl (*S*)-2-amino(pyridine-4-yl)propanoate • HCl (1:2) salt (2)

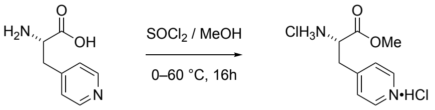

2

To a stirred solution of 3-(4-pyridyl)-L-alanine (100 mg, 0.602 mmol, 1.00 equiv.) in methanol (2.5 mL, 0.25 M) at 0 °C was added thionyl chloride (0.132 mL, 1.81 mmol, 3.00 equiv.) dropwise. The resulting reaction mixture was left to stir at 60 °C for 18 h. The reaction mixture was concentrated *in vacuo* (toxic Buchi) to give desired ester **2** as a white solid (155 mg, 100%). ^{1}H NMR (400 MHz, CD_3OD) δ_H: 3.55 (1H, dd, *J* 14.5, 6.5 Hz, 2-*H*H), 3.63 (1H, dd, *J* 14.5, 7.9 Hz, 2-H*H*), 3.83 (3H, s, CH_3), 4.67 (1H, dd, *J* 7.9, 6.5 Hz, 1-H), 8.10–8.16 (2H, m, 2 × ArH), 8.85–8.90 (2H, m, 2 × ArH); ^{13}C{^{1}H} NMR (101 MHz, CD_3OD) δ_C: 37.0 (CH_2), 53.5 (CH), 54.0 (CH_3), 129.7 (2 × CH), 142.9 (2 × CH), 158.3 (C), 169.4 (C); HRMS (ESI) $C_9H_{12}N_2O_2^+$ ([M+H$^+$]) requires 181.0972, found 181.0973 (0.6 ppm).

(*S*)-3-((*tert*-butoxycarbonyl)amino)-3-(2-methoxyphenyl)propanoic acid (3)

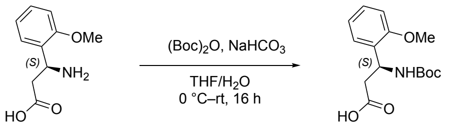

3

(*S*)-3-Amino-3-(2-methoxyphenyl)propionic acid (100 mg, 0.512 mmol, 1.00 equiv.) was dissolved in tetrahydrofuran (1.3 mL) and water (1.3 mL) and the resulting solution was cooled to 0 °C. Sodium hydrogen carbonate (129 mg, 1.54 mmol, 3.00 equiv.) was added in one portion followed by di-*tert*-butyl decarbonate. The resulting reaction mixture was left to stir at room temperature for 18 h. The tetrahydrofuran was removed under reduced pressure and the reaction mixture was acidified to pH 1 with 1 M aqueous hydrochloric acid. The aqueous layer was extracted with dichloromethane (3 × 20 mL). The combined organic layers were dried over $MgSO_4$, filtered and concentrated *in vacuo* to give the desired carbamate **3** as a white solid (131 mg, 87%); ^{1}H NMR (400 MHz, CD_3OD) δ_H: 1.42 (9H, s, 3 × CH_3), 2.63 (1H, dd, *J* 15.5, 8.8 Hz, 2-*H*H), 2.75 (1H, dd, *J* 15.5, 5.1 Hz, 2-H*H*), 3.87 (3H, s, OCH_3), 5.30 (1H, br s, 3-H), 6.90 (1H, t, *J* 7.5 Hz, ArH), 6.96 (1H, d, *J* 8.2 Hz, ArH), 7.19–7.29 (2 H, m, 2 × ArH); ^{13}C{^{1}H} NMR (101 MHz, CD_3OD) δ_C: 28.7 (3 × CH_3), 40.7 (CH_2), 54.8 (C), 55.8 (CH), 80.2 (C), 111.8 (CH), 121.5 (CH), 128.0 (CH), 129.6 (CH), 131.2 (C), 157.4 (C), 158.0 (C), 174.8 (C); HRMS (ESI) $C_{15}H_{21}NNaO_5^+$ ([M+Na$^+$]) requires 318.1312, found 318.1319 (2.2 ppm).

Ethyl (2*S*,4*S*)-1-((*S*)-3-((*tert*-butoxycarbonyl)amino)-3-(2-methoxyphenyl)propanoyl)-4-cyclohexylpyrrolidine-2-carboxylate (4)

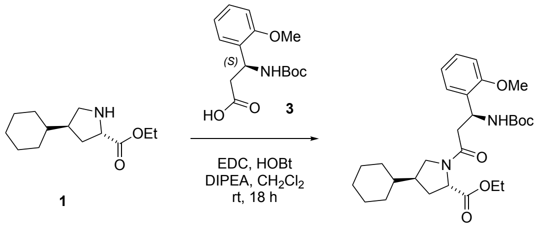

4

To a stirred solution of (*S*)-3-((*tert*-butoxycarbonyl)amino)-3-(2-methoxyphenyl)propanoic acid **3** (38 mg, 0.13 mmol, 1.0 equiv.) in dichloromethane (0.4 mL) was added *N*-(3-dimethylaminopropyl)-*N*′-ethylcarbodiimide hydrochloride (30 mg, 0.15 mmol, 1.2 equiv.) and 1-hydroxybenzotriazole hydrate (24 mg, 0.15 mmol, 1.2 equiv.) and the resulting solution was left to stir at room temperature for 10 minutes. To the reaction mixture was added a stirred solution of (2*S*,4*S*)-ethyl 4-cyclohexylpyrrolidine-2-carboxylate **1** (34 mg, 0.13 mmol, 1.0 equiv.) and *N,N*-diisopropylethylamine (0.067 mL, 0.39 mmol, 3.0 equiv.) in dichloromethane and the resulting reaction mixture was left to stir at room temperature for 18 h. The reaction mixture was concentrated *in vacuo* and purified by flash column chromatography (petroleum ether/ethyl acetate 7:3) to give desired amide **4** as a white solid (55 mg, 85%). ^{1}H NMR (400 MHz, CD_3OD) δ_H: 0.78–1.46 (19H, m), 1.54–1.91 (6H, m), 1.94–2.11 (2H, m), 2.71 (1H, dd, *J* 15.0,

5.0 Hz, 2-*H*H), 2.80 (1H, dd, *J* 15.0, 7.9 Hz, 2-H*H*), 2.88–3.03 (1H, m), 3.64–3.79 (1H, m), 3.88 (3H, s, OCH_3), 4.09–4.25 (2H, m), 4.41 (1H, d, *J* 9.3, CH), 5.34 (1H, br s, 3-H), 6.92 (1H, td, *J* 7.4, 1.1 Hz, ArH), 6.95–7.00 (1H, m, ArH), 7.20–7.29 (2H, m, 2 × ArH); $^{13}C\{^1H\}$ NMR (101 MHz, CD_3OD) δ_C: 14.4 (3 × CH_3), 27.1 (CH), 27.1 (CH), 27.4 (CH), 28.8 (CH_2), 32.3 (CH), 32.9 (CH), 34.2 (CH), 36.0 (C), 40.1 (CH), 42.9 (CH), 44.9 (CH), 51.7 (CH_2), 52.5 (CH_2), 55.9 (CH_3), 60.5 (CH), 61.4 (CH), 62.3 (CH_2), 111.7 (CH), 121.6 (CH), 127.6 (C), 129.5 (CH), 129.7 (CH), 157.9 (C), 171.9 (C), 173.7 (C); *m/z* (LC-MS, ESI^+) 503 (MH^+, 37%); HRMS (ESI) $C_{28}H_{42}N_2NaO_6^+$ ([$M+Na^+$]) requires 525.2938, found 525.2943 (0.9 ppm).

(2*S*,4*S*)-1-((*S*)-3-((*tert*-butoxycarbonyl)amino)-3-(2-methoxyphenyl)propanoyl)4-cyclohexyl-pyrrolidine-2-carboxylic acid (5)

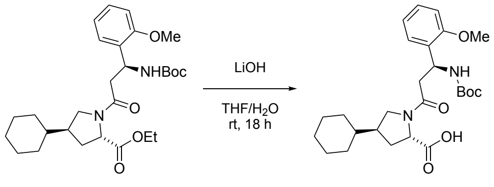

To a stirred solution of ester **4** (126 mg, 0.251 mmol, 1.00 equiv.) in tetrahydrofuran (2.4 mL) and water (2.4 mL) was added lithium hydroxide hydrate (15.8 mg, 0.376 mmol, 1.50 equiv.). The resulting reaction mixture was left to stir at room temperature for 18 h. The tetrahydrofuran was removed *in vacuo* and the reaction mixture was acidified to pH 1 with 1 M aqueous hydrochloric acid. The aqueous layer was extracted with dichloromethane (3 × 30 mL). The combined organic layers were dried over $MgSO_4$, filtered and concentrated *in vacuo* to give the desired acid **5** as a white solid (115 mg, 97%). ^1H NMR (400 MHz, CD_3OD) δ_H: 0.81–1.33 (7H, m), 1.41 (9H, s, 3 × CH_3), 1.53–1.93 (7H, m), 1.95–2.52 (2H, m), 2.63–3.02 (3H, m), 3.57–3.74 (1H, m), 3.87 (3H, s, OCH_3), 4.42 (1H, d, *J* 9.2 Hz, CH), 5.17–5.39 (1H, m, 3-H), 6.91 (1H, br t, *J* 7.4 Hz, ArH), 6.96 (1H, br d, *J* 8.5 Hz, ArH), 7.19–7.28 (2H, m, 2 × ArH); $^{13}C\{^1H\}$ NMR (101 MHz, MeOD) δ_C: 27.1, 27.1, 27.4, 28.8, 30.7, 32.3, 32.4, 32.9, 34.3, 36.0, 40.0, 40.9, 42.9, 43.0, 43.1, 44.8, 48.3, 51.7, 52.5, 54.8, 55.8, 55.9, 60.3, 61.3, 80.3, 111.6, 111.7, 121.6, 121.7, 127.6, 128.0, 129.5, 129.7, 130.9, 131.3, 157.1, 157.3, 157.8, 171.9, 171.9, 175.4; *m/z* (LC-MS, ESI^-) 473 (MH^-, 100%); HRMS (ESI) $C_{26}H_{39}N_2O_6^+$ ([$M+H^+$]) requires 475.2803, found 475.2807 (0.84 ppm).

***tert*-Butyl ((*S*)-3-((2*S*,4*S*)-4-cyclohexyl-2-(((*S*)-1-(methylamino)-1-oxo-3-(pyridine-4-yl)propan-2-yl)carbamoyl)pyrrolidin-1-yl)-1-(2-methoxyphenyl)-3-oxopropyl)carbamate (8)**

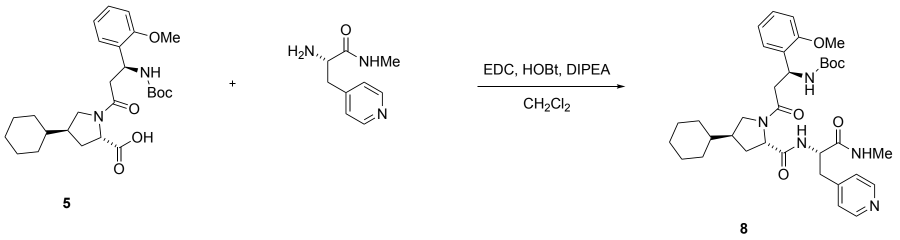

To the solution of (2*S*,4*S*)-1-((*S*)-3-((*tert*-butoxycarbonyl)amino)-3-(2-methoxyphenyl)propanoyl)-4-cyclohexylpyrrolidine-2-carboxylic acid **5** (60 mg, 0.126 mmol) in DCM (5 mL) was added DIPEA (0.066 ml, 0.379 mmol), EDC (29.1 mg, 0.152 mmol), HOBt (23.2 mg, 0.152 mmol) and stirred at RT for 2 minutes. To this reaction mixture was added (S)-2-amino-N-methyl-3-(pyridin-4-yl)propenamide (22.7 mg, 0.126 mmol) and stirred at RT for overnight. The reaction mass was concentrated purified by reverse phase column chromatography (gold column 100g snap). The product was eluted at 50% acetonitrile-water mixture and concentrated under reduced pressure to yield the desired amide **8** as an off white solid (50 mg, 0.075 mmol, 60%). ^{1}H NMR (400 MHz, CD_3OD) δ_H: 0.72–1.08 (2H, m), 1.15–1.48 (4H, m), 1.43 (9H, s, 3 × CH_3), 1.52–1.75 (7H, m), 1.84–1.89 (1H, m), 2.77–2.90 (7H, m), 3.21–3.29 (1H, m), 3.32–3.33 (1H, m), 3.67–3.69 (1H, m), 3.90 (3H, s), 4.30–4.32 (1H, m), 4.71–4.74 (1H, m), 5.44 (1H, br s), 6.93–7.02 (2H, m, 2 × ArH), 7.26–7.35 (4H, m, 4 × ArH), 8.12 (1H, br s, NH), 8.45–8.47 (2H, m, 2 × ArH); LC-MS (ESI) $C_{35}H_{50}N_5O_6^+$ ([M+H$^+$]): (t_R) 2.1 min, requires 636.4, found 636.3.

(2*S*,4*S*)-1-((*S*)-3-amino-3-(2-methoxyphenyl)propanoyl)-4-cyclohexyl-*N*-((*S*)-1-(methylamino)-1-oxo-3-(pyridin-4-yl)propan-2-yl)pyrrolidine-2-carboxamide (MRC37)

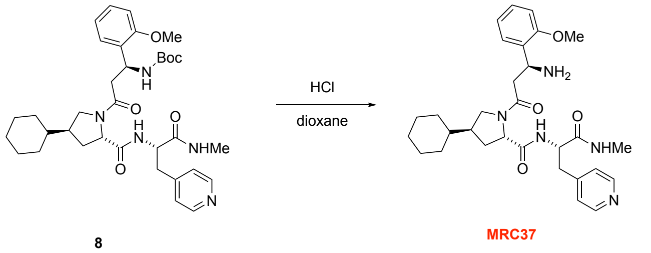

To the solution of tert-butyl ((*S*)-3-((2*S*,4*S*)-4-cyclohexyl-2-(((*S*)-1-(methylamino)-1-oxo-3-(pyridin-4-yl)propan-2-yl)carbamoyl)pyrrolidin-1-yl)-1-(2-methoxyphenyl)-3-oxopropyl)carbamate (50 mg, 0.079 mmol) in dioxane was added HCl (4M in dioxane) (0.012 mL, 0.393 mmol) and stirred at RT for overnight. The reaction mass was concentrated and purified by reverse phase column chromatography (gold column, 100 g snap). The product was eluted using 45% acetonitrile-water mixture to yield **MRC37** as green hygroscopic solid (30 mg, 0.056 mmol, 71%). ^{1}H NMR (400 MHz, DMSO-d_6) δ_H: 1.17–1.23 (2H, m), 1.26–1.31 (2H, m), 1.58–1.65 (7H, m), 2.59–2.64 (4H, m), 2.97–3.12 (5H, m), 3.31–3.61 (3H, m), 3.70–3.79 (1H, m), 3.86 (3H, s), 4.29–4.38 (1H, m), 4.62–4.78 (1H, m), 4.81–4.96 (1H, m), 6.98–7.09 (m, 1H), 7.11–7.15 (m, 1H), 7.39–7.40 (m, 1H), 7.53–7.55 (m, 1H), 7.80–7.84 (3H, m), 8.26–8.35 (3H, m), 8.76–8.78 (2H, m); LC-MS (ESI) $C_{30}H_{42}N_5O_4^+$ ([M+H$^+$]): (t_R) 1.5 min, requires 536.4, found 536.3.

III. PREPARATION OF MRC71

Methyl (*S*)-2-((2*S*,4*S*)-1-((*S*)-3-((*tert*-butoxycarbonyl)amino)-3-(2-methoxyphenyl)propanoyl-4-cyclohexylpyrrolidine-2-carboxamido)-3-(pyridin-4-yl)propanoate (6)

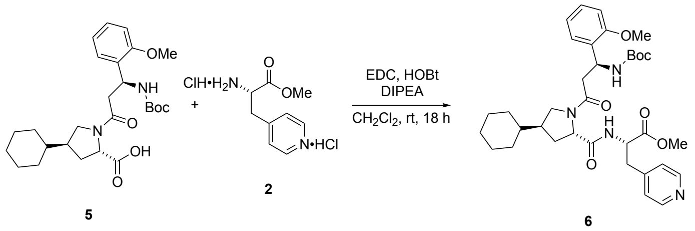

To a stirred solution of acid **5** (35 mg, 0.074 mmol, 1.0 equiv.) in dichloromethane (1.0 mL) was added *N*-(3-dimethylaminopropyl)-*N'*-ethylcarbodiimide hydrochloride (17 mg, 0.089 mmol, 1.2 equiv.) and 1-hydroxybenzotriazole hydrate (14 mg, 0.089 mmol, 1.2 equiv.) and the resulting solution was left to stir at room temperature for 10 minutes. To the reaction mixture was added a stirred solution of amine **2** (16 mg, 0.074 mmol, 1.0 equiv.) and *N,N*-diisopropylethylamine (0.039 mL, 0.22 mmol, 3.0 equiv.) in dichloromethane and the resulting reaction mixture was left to stir at room temperature for 18 h. The reaction mixture was concentrated *in vacuo* and purified by flash column chromatography (dichloromethane/methanol 49:1) to give desired product **6** as a white solid (32 mg, 79%). ^{1}H NMR (400 MHz, $CDCl_3$) δ_H: 0.66–1.22 (7H, m), 1.41 (9H, s, 3 × CH_3), 1.54–1.81 (5H, m), 2.18–2.36 (2H, m, 2-H_2), 2.59 (1H, dd, *J* 14.5, 5.2 Hz), 2.78–2.96 (2H, m), 3.08–3.23 (2H, m), 3.74 (3H, s, OCH_3), 3.87 (3H, s, OCH_3), 4.42 (1 H, d, *J* 7.9 Hz, CH), 4.82 (1H, td, *J* 8.3, 5.4 Hz), 5.36 (1H, br s), 6.33–6.45 (1H, m, NH), 6.80–6.94 (2H, m, ArH), 6.98–7.08 (2H, m, 2 × ArH), 7.17–7.29 (2H, m, 2 × ArH), 7.61 (1H, br d, *J* 8.2 Hz, NH), 8.43–8.59 (2H, m, 2 × ArH); $^{13}C\{^{1}H\}$ NMR (101 MHz, $CDCl_3$) δ_C: 26.0 (CH_2), 26.1 (CH_2), 26.3 (CH_2), 28.5 (3 × CH_3), 30.9 (CH_2), 31.3 (CH_2), 31.9 (CH_2), 37.4 (CH_2), 38.3 (CH_2), 41.9 (CH), 43.8 (CH), 47.3 (CH), 51.7 (CH_2), 52.3 (CH), 52.6 (CH_3), 55.5 (CH_3), 59.8 (CH), 77.4 (C), 79.5 (C), 110.4 (CH), 110.6 (C), 120.8 (CH), 124.7 (2 × CH), 127.3 (CH), 128.6 (CH), 145.7 (C), 149.7 (2 × CH), 155.1 (C), 156.4 (C), 170.9 (C), 171.4 (C); LC-MS (ESI) $C_{35}H_{49}N_4O_7^+$ ([M+H$^+$]) found 637.2 (100%); HRMS (ESI) $C_{35}H_{48}N_4NaO_7^+$ ([M+Na$^+$]) requires 659.3415, found 659.3419 (1.1 ppm).

(*S*)-2-((2*S*,4*S*)-1-((*S*)-3-((*tert*-butoxycarbonyl)amino)-3-(2-methoxyphenyl)propanoyl-4-cyclohexylpyrrolidine-2-carboxamido)-3-(pyridin-4-yl)propanoic acid (7)

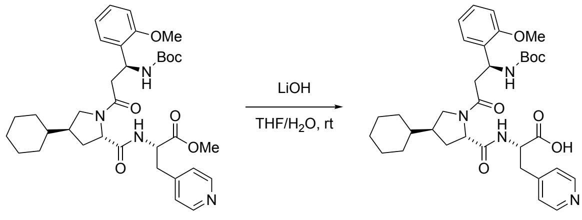

To a stirred solution of ester **6** (30 mg, 0.047 mmol, 1.0 equiv.) in tetrahydrofuran (0.5 mL) and water (0.5 mL) was added lithium hydroxide hydrate (3.0 mg, 0.071 mmol, 1.5 equiv.). The resulting reaction mixture was left to stir at room temperature for 4 h. The tetrahydrofuran was removed *in vacuo* and the reaction mixture was acidified to pH 1 with 1 M aqueous hydrochloric acid. The aqueous layer was extracted with dichloromethane (3 × 20 mL). The combined organic layers were dried over $MgSO_4$, filtered and concentrated *in vacuo* to give the desired acid **7** as a white solid (20 mg, 69%). ^{1}H NMR (400 MHz, CD_3OD) δ_H: 0.71–2.10 (25H, m), 2.29–2.71 (2H, m), 2.74–3.15 (3H, m), 3.36–3.77 (2H, m), 3.85 (3H, s, OCH_3), 4.37 (1H, d, *J* 8.8 Hz), 4.69 (1H, dd, *J* 8.1, 5.0 Hz), 5.25–5.42 (1H, m), 6.84–7.01 (2H, m), 7.17–7.33 (3H, m), 7.39 (1H, d, *J* 5.1 Hz), 8.26–8.52 (2H, m); ^{13}C{^{1}H} NMR (101 MHz, CD_3OD) δ_C: 25.7, 25.7, 26.0, 27.4, 31.0, 31.6, 33.0, 35.4, 36.4, 38.5, 41.6, 43.3, 50.7, 51.4, 54.6, 60.0, 61.3, 110.3, 110.4, 120.2, 124.8, 125.5, 128.2, 147.8, 148.5, 149.0, 156.4, 156.5, 170.7, 172.7; LC-MS (ESI) $C_{34}H_{47}N_4O_7^+$ ([M+H$^+$]) 623.8 (100%); HMRS (APCI) $C_{34}H_{47}N_4O_7^+$ ([M+H$^+$]) requires 623.3439, found 623.3439.

***tert*-Butyl ((*S*)-3-((2*S*,4*S*)-2-(((*S*)-22-chloro-3-oxo-1-(pyridin-4-yl)-7,10,13,16-tetraoxa-3-azadocosan-2-yl)carbamoyl)4-cyclohexylpyrrolidin-1-yl)-1-(2-methoxyphenyl)-3-oxopropyl)-carbamate (9)**

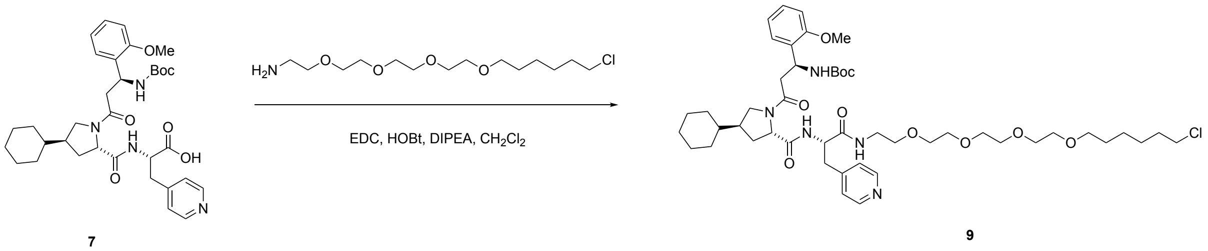

To the solution of (*S*)-2-((2*S*,4*S*)-1-((*S*)-3-((*tert*-butoxycarbonyl)amino)-3-(2-methoxyphenyl)propanoyl)-4-cyclohexylpyrrolidine-2-carboxamido)-3-(pyridin-4-yl)propanoic acid (35 mg, 0.056 mmol) in DCM (2 mL) was added EDC (13 mg, 0.067 mmol), HOBt (10 mg, 0.067 mmol), DIPEA (0.029 mL, 0.17 mmol) and stirred at 0 °C for 5 minutes. To this reaction mixture was added 18-chloro-3,6,9,12-tetraoxaoctadecan-1-amine (18 mg, 0.056 mmol) and stirred at RT overnight. The

progress of the reaction was monitored by LCMS which indicated the product mass along with the starting material. Therefore 18-chloro-3,6,9,12-tetraoxaoctadecan-1-amine (18 mg, 0.056 mmol), EDC (13 mg, 0.067 mmol), HOBt (10 mg, 0.067 mmol) and DIPEA (0.029 mL, 0.17 mmol) were added. The reaction mixture was again continued to stir at RT for overnight. Reaction mass was concentrated under reduced pressure and purified by reverse phase column chromatography (40g snap gold column). Product fractions were eluted at 69% acetonitrile-water mixture. The obtained fractions were concentrated and dried to yield the desired amide 9 as a yellow gum (19 mg, 0.021 mmol, 37 % yield). A sample was analysed by LCMS, which indicated the desired product mass. The product was taken forward without further characterization. LC-MS (APCI) $C_{48}H_{75}ClN_5O_{10}$ ($[M+H^+]$): (t_R) 2.55 min, requires 916.5, found 916.5.

(2*S*,4*S*)-1-((*S*)-3-amino-3-(2-methoxyphenyl)propanoyl)-*N*-((*S*)-22-chloro-3-oxo-1-(pyrridin-4-yl)-7,10,13,16-tetraoxa-4-azadocosan-2-yl)-4-cyclohexylpyrrolidine-2-carboxamide (MRC71)

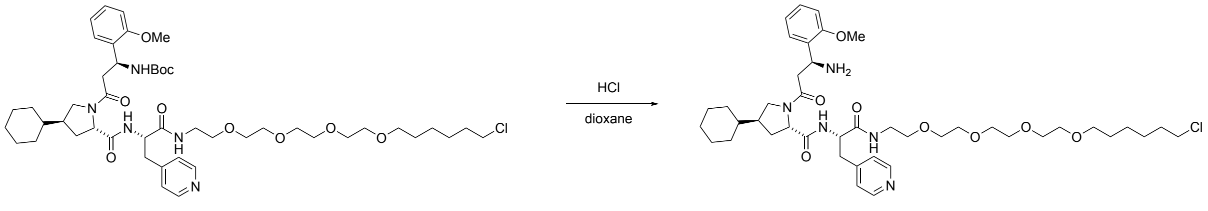

9

MRC71

To the solution of *tert*-butyl-((*S*)-3-((2*S*,4*S*)-2-(((*S*)-22-chloro-3-oxo-1-(pyridin-4-yl)-7,10,13,16-tetraoxa-4-azadocosan-2-yl)carbamoyl)-4-cyclohexylpyrrolidin-1-yl)-1-(2-methoxyphenyl)-3-oxopropyl)carbamate (19 mg, 0.021 mmol) in DCM (1 mL) was added HCl (4M in dioxane) (5.2 µL, 0.021 mmol) at 0 °C and the reaction mixture was stirred at RT for overnight. The reaction mass was concentrated under reduced pressure and further dried by lyophilization to yield **MRC71**•2HCl as a white solid (15 mg, 0.016 mmol, 79% yield). ^1H NMR (400 MHz, CD_3OD) δ_H: 0.87–1.07 (3H, m), 1.12–1.51 (11H, m), 1.53–1.66 (2H, m), 1.65–1.86 (8H, m), 1.92–2.09 (2H, m), 3.04–3.27 (3H, m), 3.42–3.51 (5H, m), 3.53–3.75 (18H, m), 3.79–3.87 (1H, m), 3.97 (s, 3H), 4.43–4.53 (1H, m), 4.98–5.02 (2H, m), 7.02–7.08 (1H, m), 7.16–7.19 (1H, m), 7.38–7.51 (2H, m), 8.02–8.17 (3H, m), 8.81 (2H, br s); LC-MS (APCI) $C_{43}H_{66}ClN_5O_8$ ($[M+H^+]$): (t_R) 2.7 min, requires 816.5, found 816.5.

IV. PREPARATION OF 414 dTAG-TRIMTAC

(*R*)-3-(3,4-dimethoxyphenyl)-1-(2-((2,2-dimethyl-4,20-dioxo-3,9,12,15-tetraoxa-5,19-diazahenicosan-21-yl)oxy)phenyl)propyl (*S*)-1-((*S*)-2-(3,4,5-trimethoxyphenyl)butanoyl)-piperidine-2-carboxylate (10)

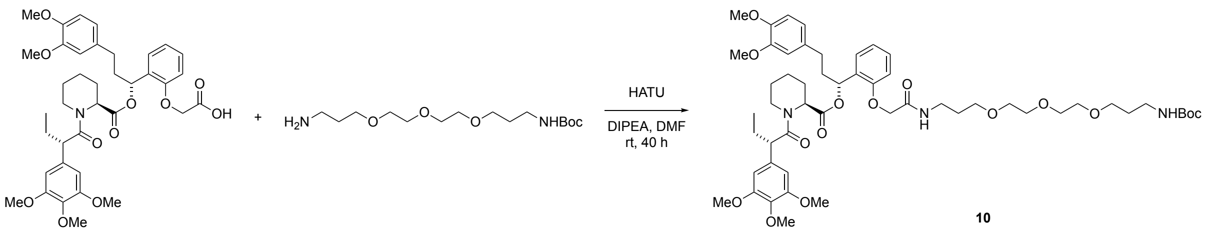

To a stirred solution of dTAG acid (15 mg, 0.022 mmol, 1.0 equiv.) and *N*-Boc-4,7,10-trioxa-1,13-tridecanediamine (6.9 mg, 0.022 mmol, 1.0 equiv.) in *N,N*-dimethylformamide (0.10 mL) was added a stirred solution of 1-[*bis*(dimethylamino)methylene]-1*H*-1,2,3-triazolo[4,5-*b*]pyridinium 3-oxid hexafluorophosphate (8.2 mg, 0.022 mmol, 1.0 equiv.) and *N,N*-diisopropylethylamine (0.011 mL, 0.065 mmol, 3.0 equiv.) in *N,N*-dimethylformamide (0.1 mL). The resulting reaction mixture was left to stir at room temperature in the dark for 18 h. Due to the remaining presence of **1** by TLC and NMR, a further portion of *N*-Boc-4,7,10-trioxa-1,13-tridecanediamine (2.0 mg) and 1-[*bis*(dimethylamino)methylene]-1*H*-1,2,3-triazolo[4,5-*b*]pyridinium 3-oxid hexafluorophosphate (2.0 mg) were added to the reaction mixture and this was left to stir for a further 24 h. The reaction mixture was washed with 5% aqueous lithium chloride and extracted with ethyl acetate (3 × 20 mL). The combined organic layers were washed with 5% aqueous lithium chloride (2 × 20 mL), dried over $MgSO_4$, filtered and concentrated *in vacuo*. The residue was purified by flash column chromatography (0–5% methanol in dichloromethane) to give desired amide **10** as a yellow oil (16 mg, 74%). ^{1}H NMR (400 MHz, CD_3OD) δ_H: 0.88 (3H, t, *J* 7.3 Hz, 1-H), 1.16–1.48 (11H, m), 1.55–1.83 (8H, m), 1.87–2.15 (3H, m), 2.27 (1H, d, *J* 13.2 Hz), 2.40–2.68 (3H, m), 3.06–3.29 (4H, m), 3.35–3.63 (13H, m) , 3.64–3.84 (15H, m), 3.87 (1H, t, *J* 7.3 Hz), 4.13 (1H, br d, *J* 13.7 Hz), 4.38–4.68 (2H, m), 5.38–5.48 (1H, m), 6.13 (1H, dd, *J* 8.2, 5.8 Hz), 6.32–7.50 (10 H, m); ^{13}C{^{1}H} NMR (101 MHz, CD_3OD) δ_C: 12.6, 21.9, 26.4, 27.6, 28.8, 29.3, 30.3, 30.9, 32.2, 37.6, 38.9, 45.0, 51.0, 53.4, 56.5, 56.5, 56.6, 56.7, 61.1, 61.1, 68.3, 69.8, 69.9, 70.8, 71.2, 71.5, 79.8, 106.6, 113.0, 113.1, 113.5, 113.6, 121.7, 121.8, 123.2, 128.3, 130.0, 130.5, 135.0, 136.9, 138.0, 148.8, 150.4, 154.6, 155.5, 170.6, 172.5, 174.8; HRMS (ESI) $C_{53}H_{77}N_3NaO_{15}^+$ ([M+Na$^+$]) requires 1018.5247, found 1018.5265 (1.8 ppm).

(*R*)-1-(2-((16-amino-2-oxo-7,10,13-trioxa-3-azahexadecyl)oxy)phenyl)-3-(3,4-dimethoxyphenyl)-propyl (*S*)-1-((*S*)-2-(3,4,5-trimethoxyphenyl)butanoyl)piperidine-2-carboxylate hydrochloride (11)

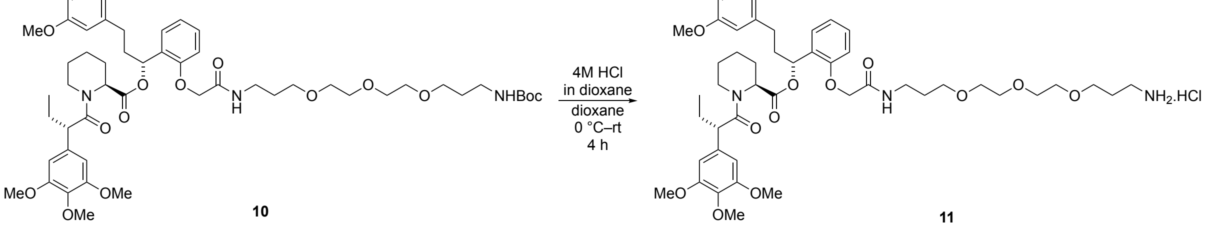

To a stirred solution of carbamate **10** (16 mg, 0.016 mmol, 1.00 equiv.) in 1,4-dioxane (0.20 mL) at 0 °C was added 4 M hydrochloric acid in 1,4-dioxane (0.20 mL). The resulting reaction mixture was left to stir at room temperature for 4 h. The reaction mixture was concentrated *in vacuo* to give desired amine **11** as a colourless oil (14 mg, 93%). The product was carried forward to the next step without further purification or characterisation.

(*R*)-1-(2-(((*S*)-1-((2*S*,4*S*)-1-((*S*)-3-((*tert*-butoxycarbonyl)amino)-3-(2-methoxyphenyl)propanoyl)-4-cyclohexylpyrrolidin-2-yl)-1,4,20-trioxo-3-(pyridin-4-ylmethyl)-9,12,15-trioxa-2,5,19-triazahenicosan-21-yl)oxy)phenyl)-3-(3,4-dimethoxyphenyl)propyl (*S*)-1-((*S*)-2-(3,4,5-trimethoxyphenyl)butanoyl)piperidine-2-carboxylate (12)

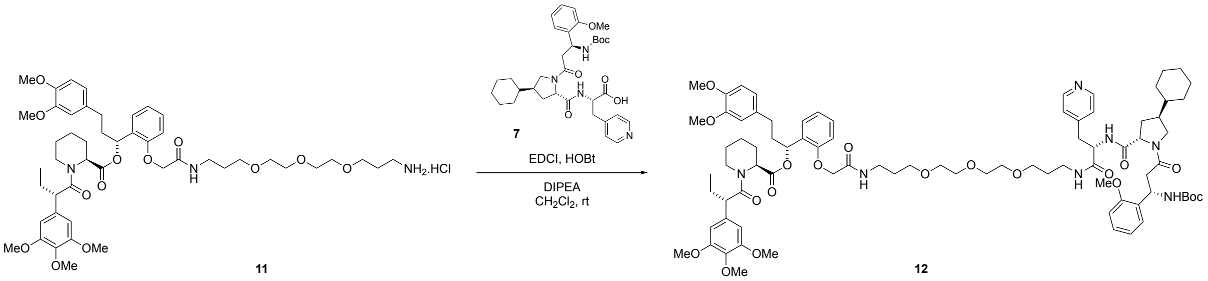

To a stirred solution of acid **7** (4.0 mg, 0.0064 mmol, 1.0 equiv.) in dichloromethane (0.50 mL) was added *N*-(3-dimethylaminopropyl)-*N*'-ethylcarbodiimide hydrochloride (1.5 mg, 0.0077 mmol, 1.2 equiv.) and 1-hydroxybenzotriazole hydrate (1.2 mg, 0.0077 mmol, 1.2 equiv.) and the resulting solution was left to stir at room temperature for 10 minutes. To the reaction mixture was added a stirred solution of amine **11** (6.0 mg, 0.0064 mmol, 1.0 equiv.) and *N,N*-diisopropylethylamine (0.0025 mL, 0.019 mmol, 3.0 equiv.) in dichloromethane (0.50 mL) and the resulting reaction mixture was left to stir at room temperature for 20 h. The reaction mixture was concentrated *in vacuo* and purified by flash column chromatography (0–5% methanol in dichloromethane) to give the desired product **12** as a colourless oil (4 mg, 41%). ^{1}H NMR (400 MHz, CD_3OD) δ_H: 0.68–3.08 (51H, m), 3.10–3.26 (3H, m), 3.35–3.93 (31H, m), 3.95–4.74 (6H, m), 5.27–5.47 (2H, m), 6.13 (1H, t, *J* 7.0 Hz), 6.32–7.13 (11H, m), 7.14–7.87 (6H, m), 8.42 (2H, d, *J* 4.6 Hz); LC-MS (ESI) $C_{82}H_{113}N_7O_{19}$ ([M-Boc+2H]$^{2+}$) 701.1 (38%).

(*R*)-1-(2-(((*S*)-1-((2*S*,4*S*)-1-((*S*)-3-amino-3-(2-methoxyphenyl)propanoyl)-4-cyclohexylpyrrolidin-2-yl)-1,4,20-trioxo-3-(pyridin-4-ylmethyl)-9,12,15-trioxa-2,5,19-triazahenicosan-21-yl)oxy)phenyl)-3-

(3,4-dimethoxyphenyl)propyl (*S*)-1-((*S*)-2-(3,4,5-trimethoxyphenyl)butanoyl)piperidine-2-carboxylate (414 dTAG-TRIMTAC)

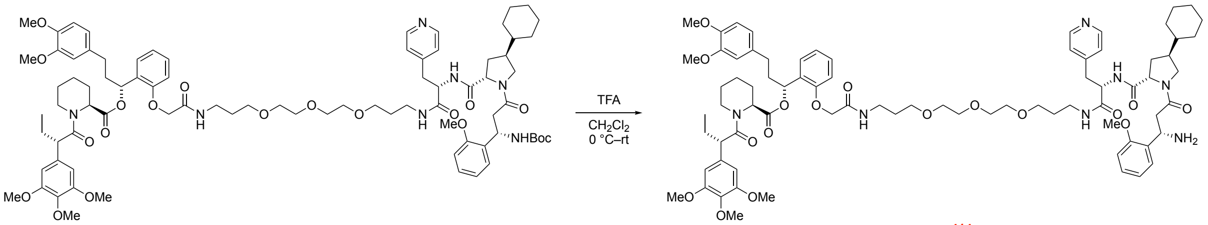

12

414
dTAG-TRIMTAC

To a stirred solution of carbamate **12** (4.0 mg, 0.0027 mmol, 1.0 equiv.) in dichloromethane (0.10 mL) at 0 °C was added trifluoroacetic acid (0.0020 mL, 0.027 mmol, 10 equiv.) as a stock solution in dichloromethane. The resulting reaction mixture was left to stir at room temperature for 2 h. The reaction mixture was concentrated *in vacuo* to give desired amine **414 (dTAG-TRIMTAC)** as a colourless oil (4.0 mg, quant.). To prepare the compound for biological evaluation, the product was further purified by preparative HPLC (30–95% acetonitrile (+ 0.1% formic acid) in water (+ 0.1% formic acid) – desired product eluted as the di formic acid salt at tR = 13 minutes (48% acetonitrile)) and lyophilised to afford **414 (dTAG-TRIMTAC)** as a white solid (2.0 mg). ^{1}H NMR (400 MHz, CD_3OD) δ_H: 0.69–0.97 (6H, m), 0.99–1.31 (7H, m), 1.42–1.83 (16H, m), 1.86–2.12 (5H, m), 2.26 (1H, d, *J* 12.5 Hz), 2.39–2.70 (4H, m), 2.78–3.15 (6H, m), 3.13 (1H, p, *J* 1.6 Hz), 3.21–3.28 (4H, m), 3.36–3.42 (2H, m), 3.43–3.61 (11H, m), 3.64–3.71 (7H, m), 3.75 (1H, d, *J* 7.0), 3.77–3.82 (7H, m), 3.82–3.91 (4H, m), 4.14 (1H, br d, *J* 13.5 Hz), 4.37–4.50 (2H, m), 4.52–4.77 (3H, m), 5.42 (1H, s), 6.13 (1H, dd, *J* 8.2, 5.7 Hz), 6.54–6.65 (2H, m), 6.67 (1H, dd, *J* 8.2, 2.0 Hz), 6.73–6.80 (1H, m), 6.81–6.93 (2H, m), 6.94–7.11 (2H, m), 7.20–7.50 (4H, m), 8.37–8.49 (2H, m), 8.54 (1H, br s); LC-MS (ESI) $C_{77}H_{105}N_7O_{17}$ ([M+2H]$^{2+}$) 701.1 (100%).

V. SPECTRAL DATA

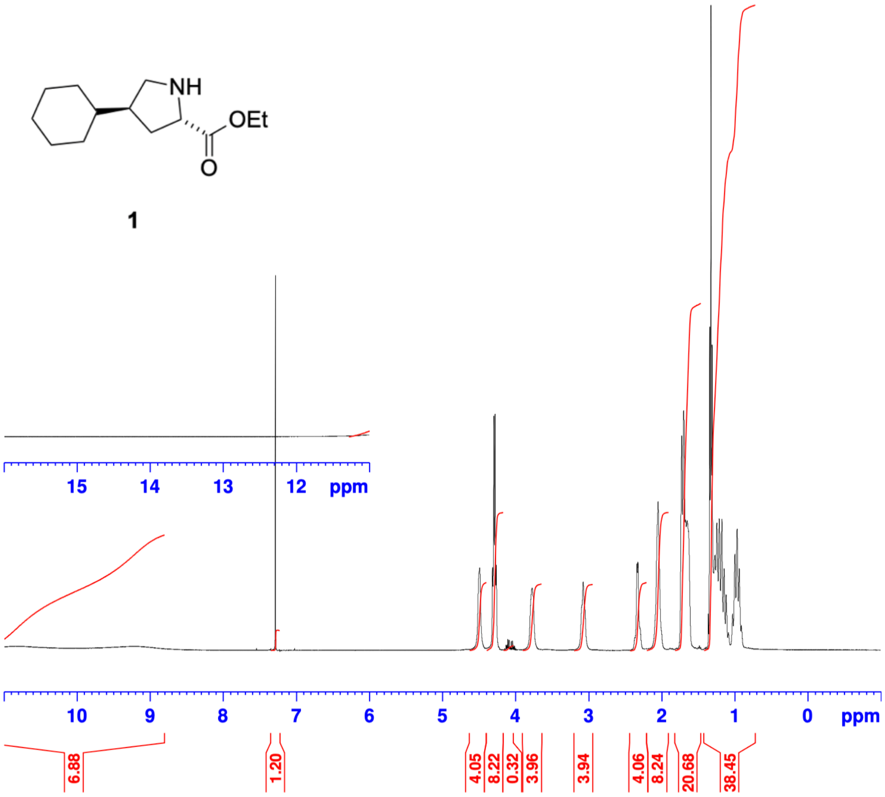

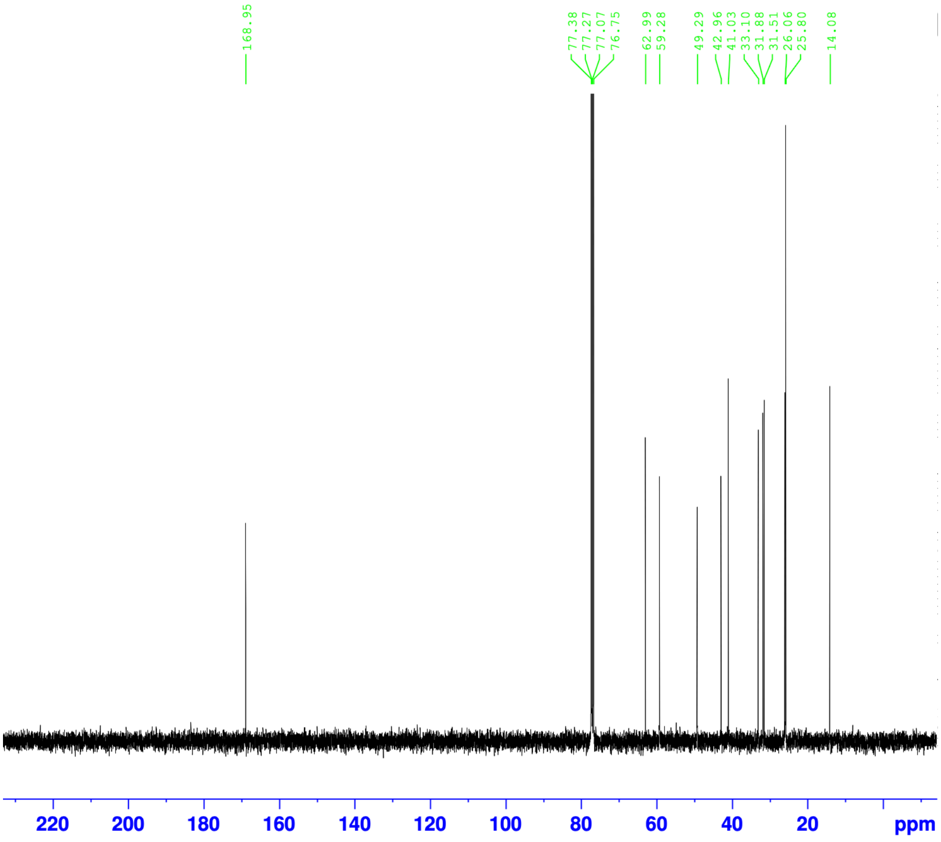

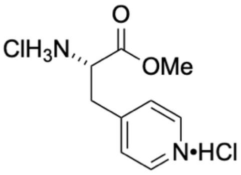
O
ClH3N
OMe
N•HCl

2

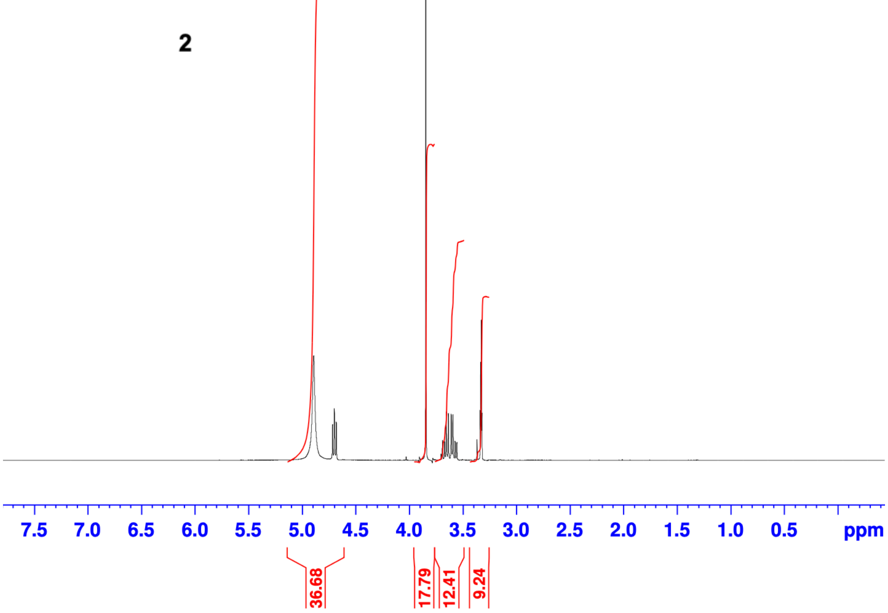
7.5
7.0
6.5
6.0
5.5
5.0
4.5
4.0
3.5
3.0
2.5
2.0
1.5
1.0
0.5
ppm
36.68
17.79
12.41
9.24

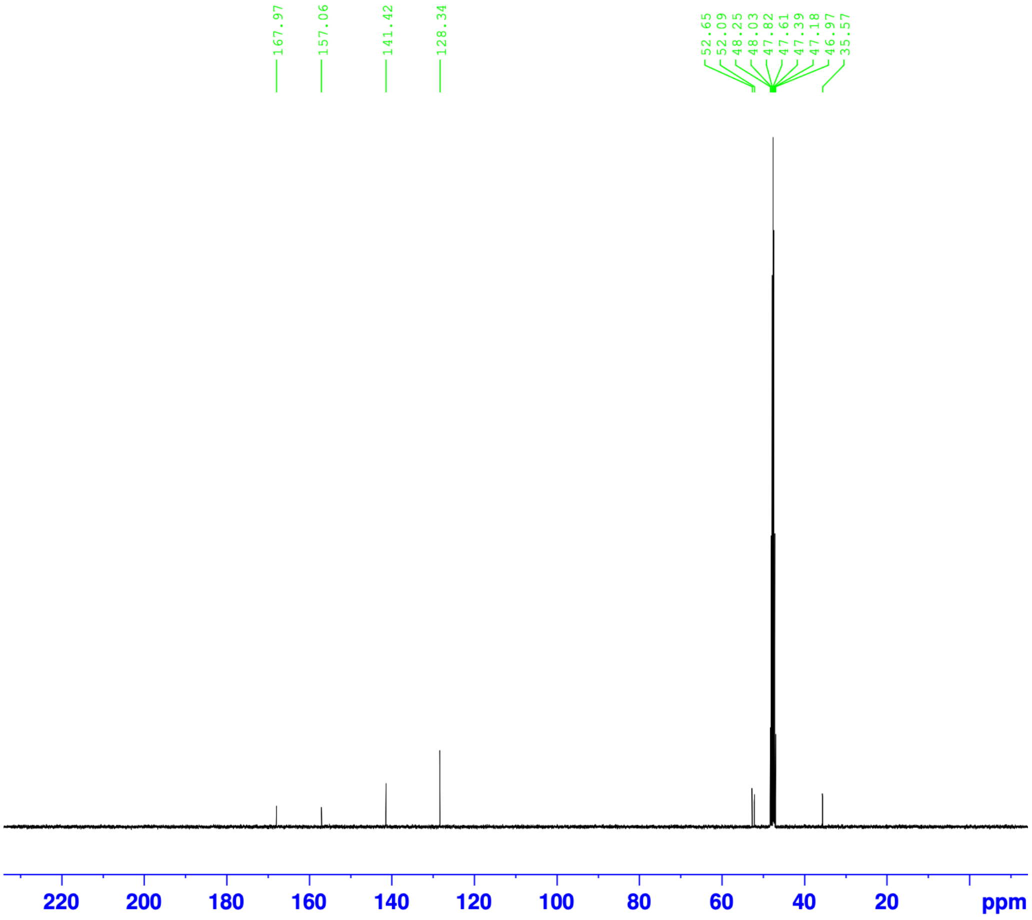
167.97
157.06
141.42
128.34
52.65
52.09
48.25
48.03
47.82
47.61
47.39
47.18
46.97
35.57
220
200
180
160
140
120
100
80
60
40
20
ppm

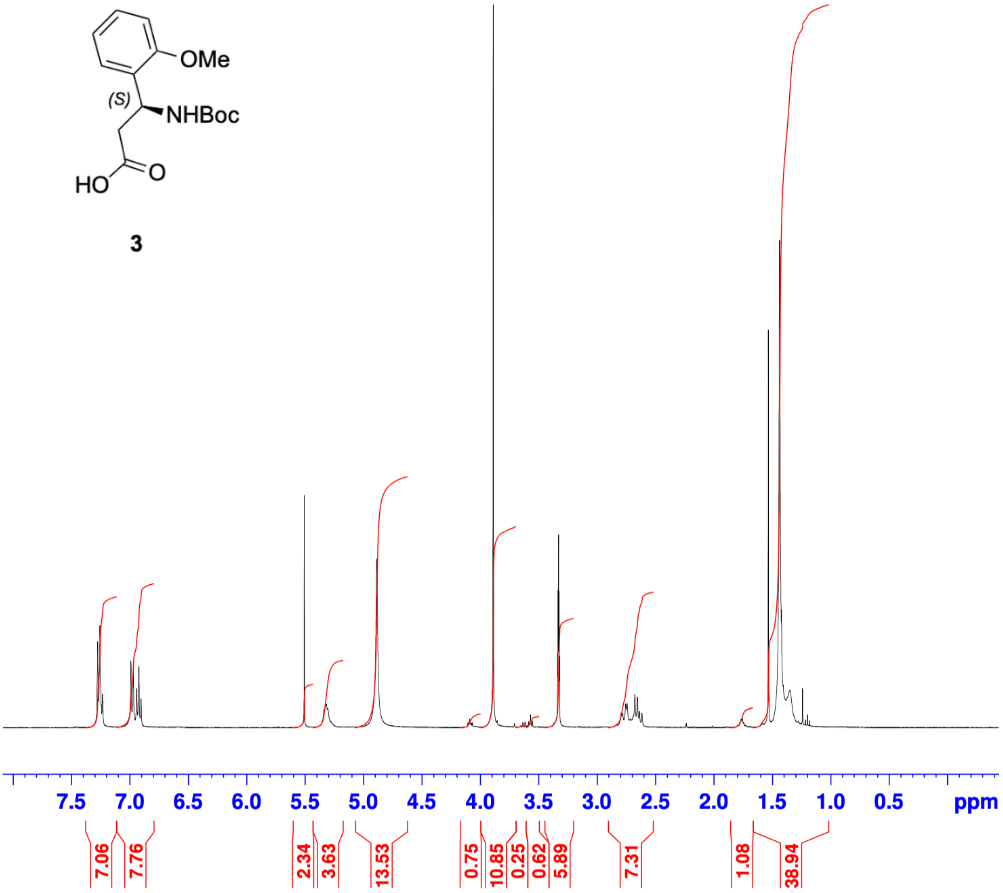
OMe
(S)
NHBoc
HO
O
3
7.5 7.0 6.5 6.0 5.5 5.0 4.5 4.0 3.5 3.0 2.5 2.0 1.5 1.0 0.5 ppm
7.06 7.76 2.34 3.63 13.53 0.75 10.85 0.25 0.62 5.89 7.31 1.08 38.94

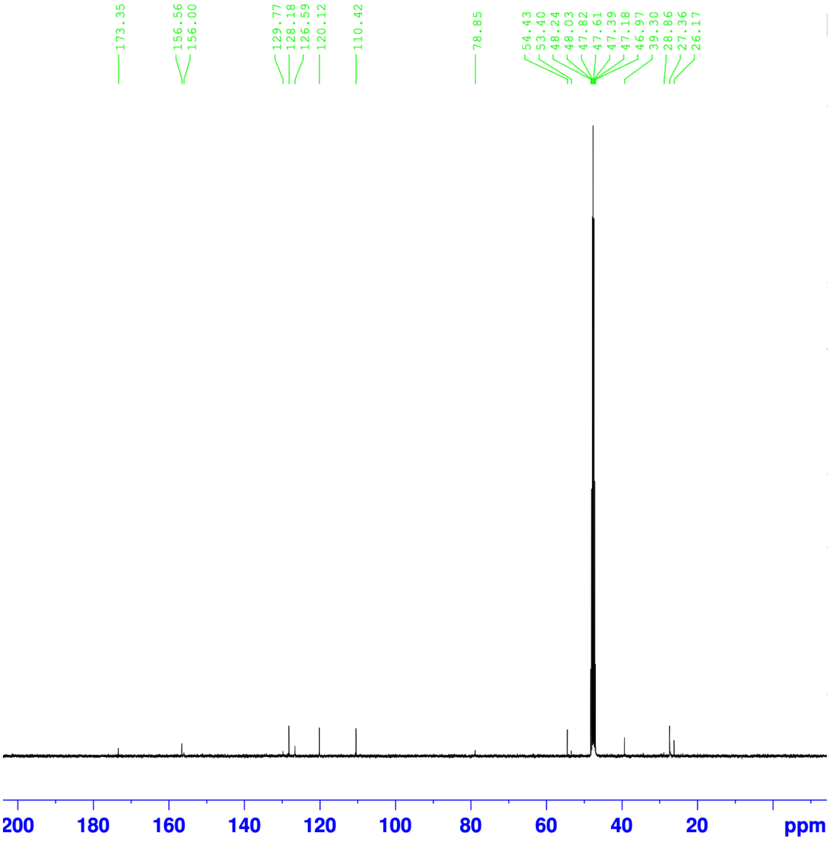
173.35 156.56 156.00 129.77 128.18 126.59 120.12 110.42 78.85 54.43 53.40 48.24 48.03 47.82 47.61 47.39 47.18 46.97 39.30 28.86 27.36 26.17
200 180 160 140 120 100 80 60 40 20 ppm

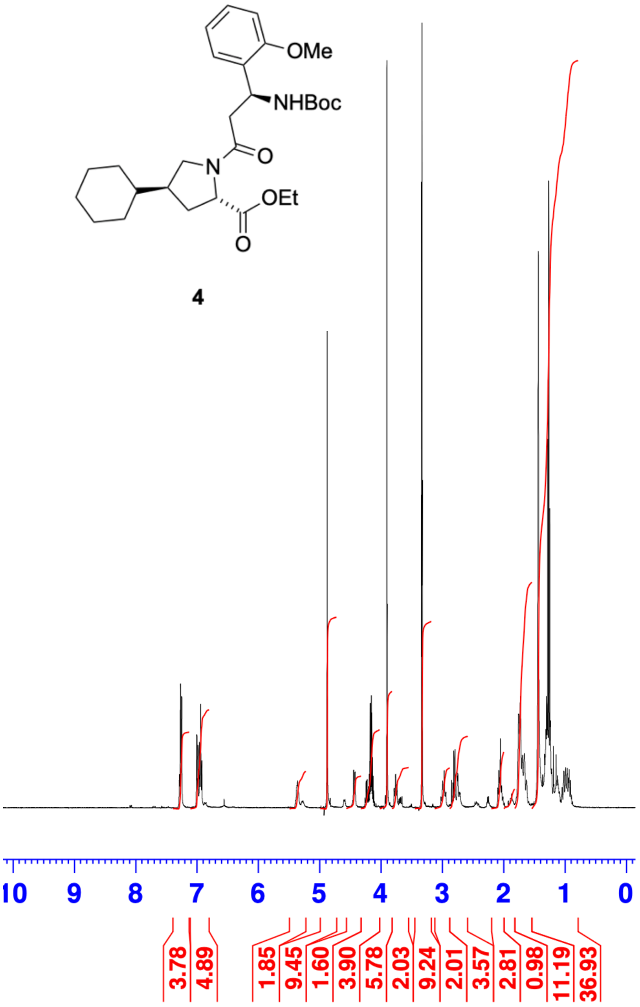
OMe
NHBoc
N
O
OEt
O
4
10 9 8 7 6 5 4 3 2 1 0
3.78
4.89
1.85
9.45
1.60
3.90
5.78
2.03
9.24
2.01
3.57
2.81
0.98
11.19
36.93

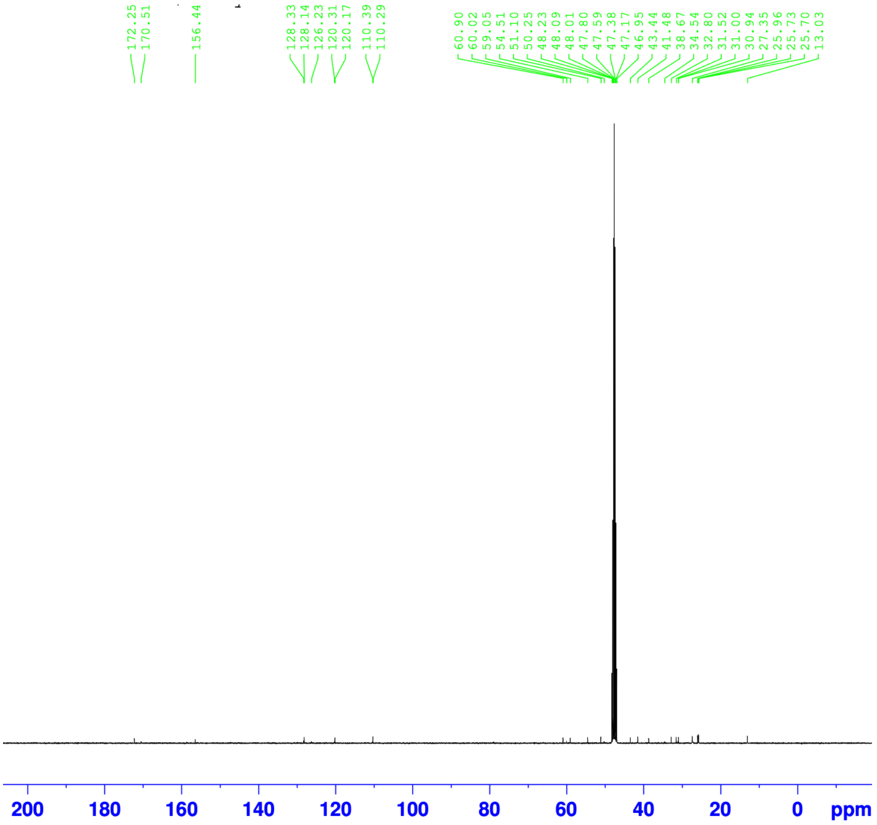
172.25
170.51
156.44
128.33
128.14
126.23
120.31
120.17
110.39
110.29
60.90
60.02
59.05
54.51
51.10
50.25
48.23
48.09
48.01
47.80
47.59
47.38
47.17
46.95
43.44
41.48
38.67
34.54
32.80
31.52
31.00
30.94
27.35
25.96
25.73
25.70
13.03
200 180 160 140 120 100 80 60 40 20 0 ppm

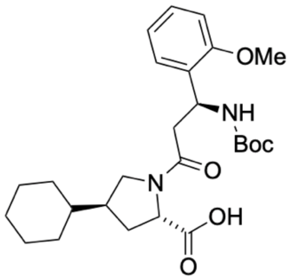
OMe
NH
Boc
N
O
OH
O

5

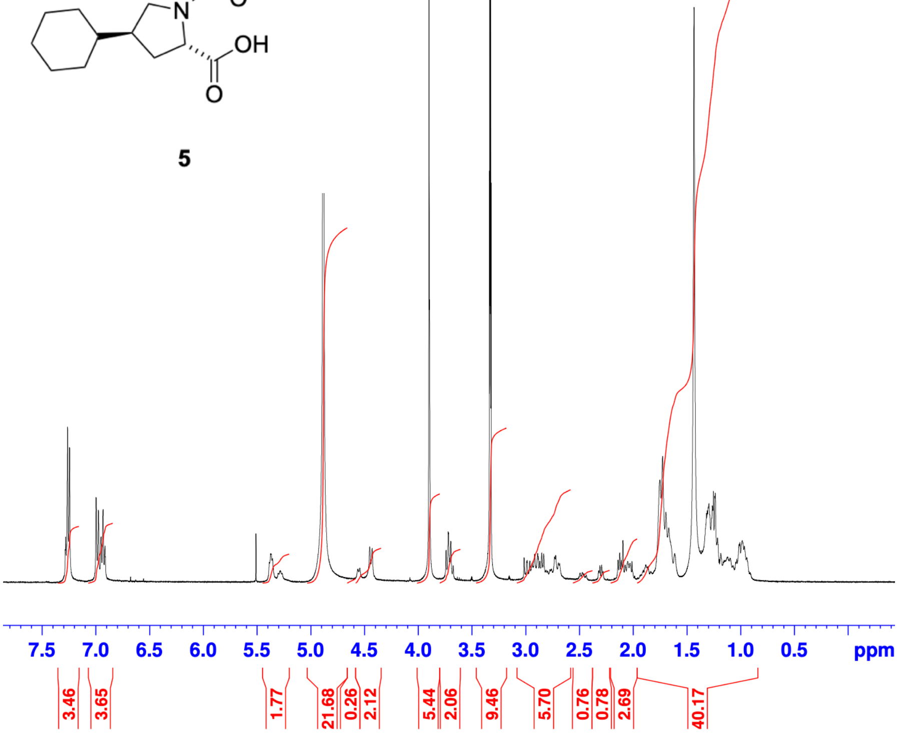
7.5 7.0 6.5 6.0 5.5 5.0 4.5 4.0 3.5 3.0 2.5 2.0 1.5 1.0 0.5 ppm
3.46 3.65 1.77 21.68 0.26 2.12 5.44 2.06 9.46 5.70 0.76 0.78 2.69 40.17

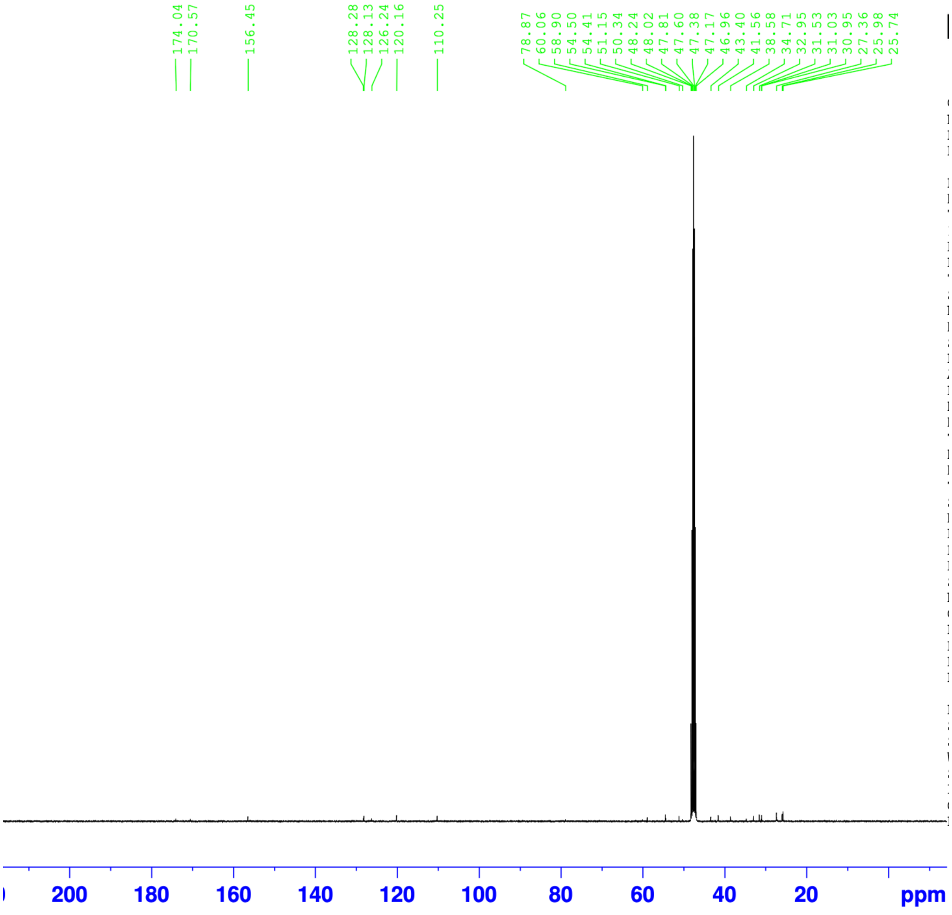
174.04 170.57 156.45 128.28 128.13 126.24 120.16 110.25 78.87 60.06 58.90 54.50 54.41 51.15 50.34 48.24 48.02 47.81 47.60 47.38 47.17 46.96 43.40 41.56 38.58 34.71 32.95 31.53 31.03 30.95 27.36 25.98 25.74
200 180 160 140 120 100 80 60 40 20 ppm

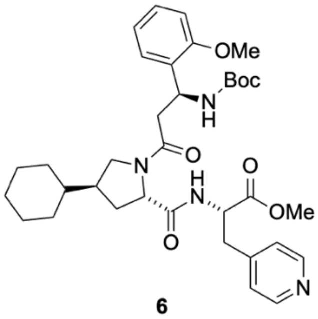
OMe
Boc
N
H
N
O
H
N
O
OMe
O
N

6

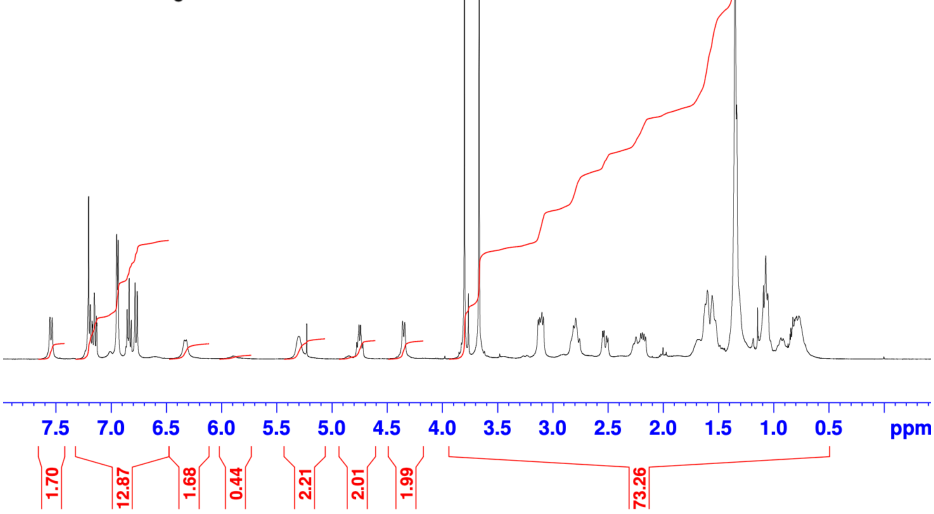
7.5
7.0
6.5
6.0
5.5
5.0
4.5
4.0
3.5
3.0
2.5
2.0
1.5
1.0
0.5
ppm
1.70
12.87
1.68
0.44
2.21
2.01
1.99
73.26

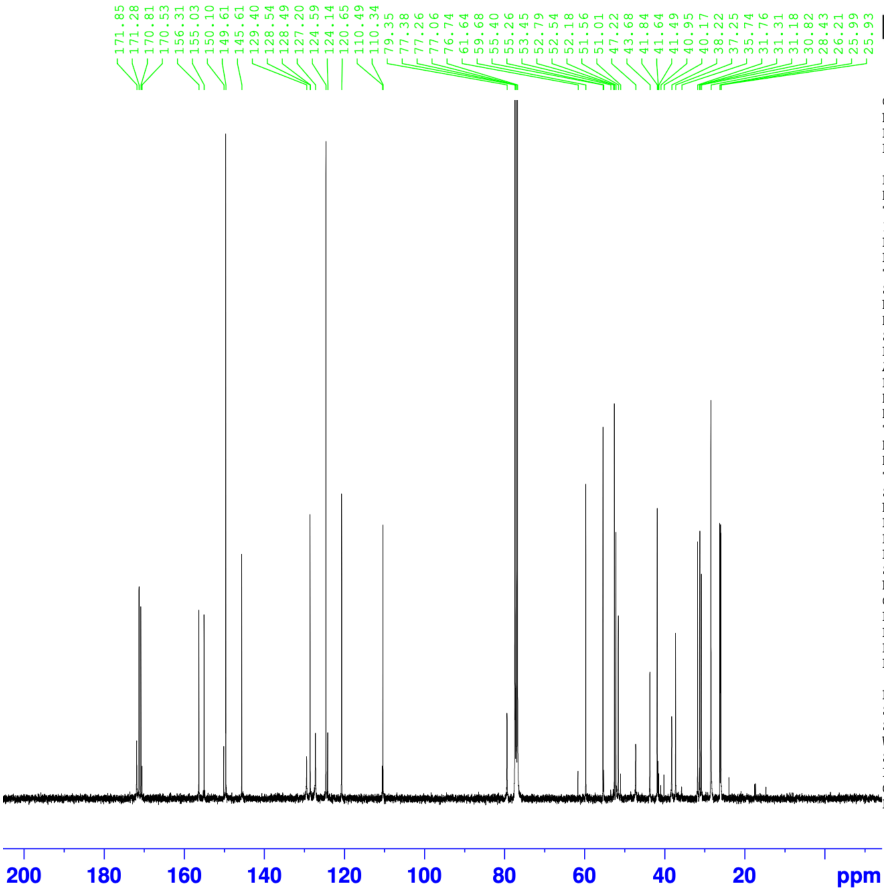
171.85
171.28
170.81
170.53
156.31
155.03
150.10
149.61
145.61
129.40
128.54
128.49
127.20
124.59
124.14
120.65
110.49
110.34
79.35
77.38
77.26
77.06
76.74
61.64
59.68
55.40
55.26
53.45
52.79
52.54
52.18
51.56
51.01
47.22
43.68
41.84
41.64
41.49
40.95
40.17
38.22
37.25
35.74
31.76
31.31
31.18
30.82
28.43
26.21
25.99
25.93
200
180
160
140
120
100
80
60
40
20
ppm

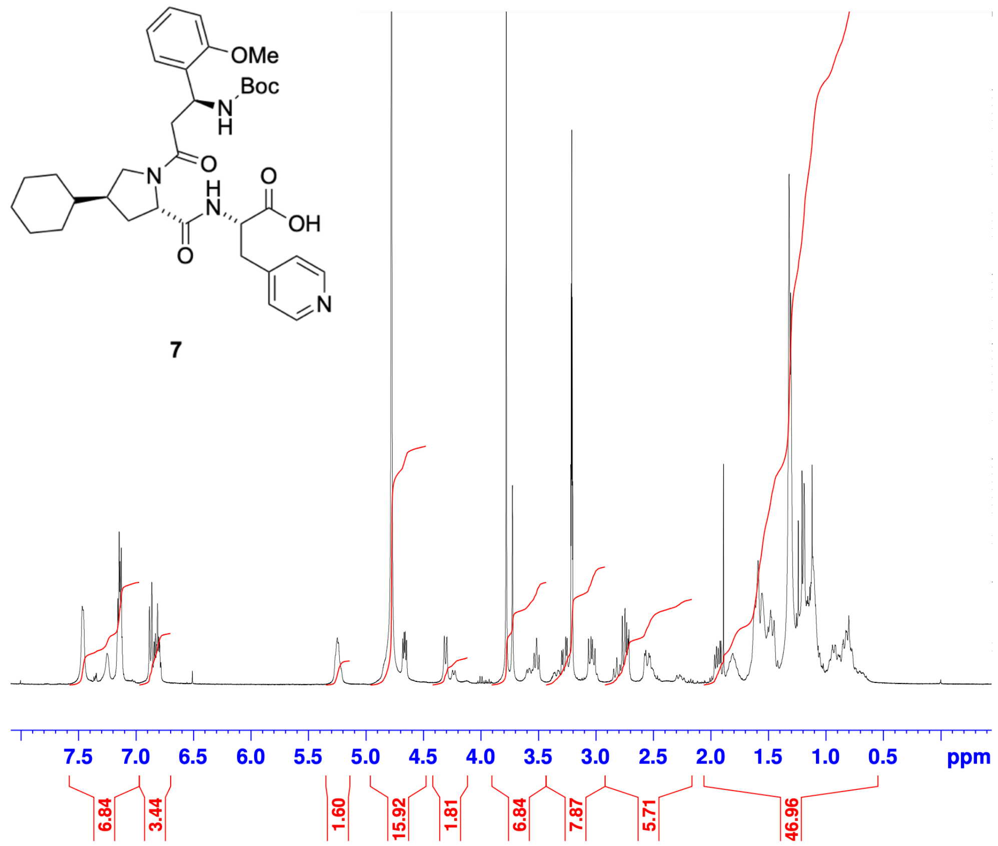
OMe
Boc
N
H
N
O
H
N
O
OH
O
N
7
7.5
7.0
6.5
6.0
5.5
5.0
4.5
4.0
3.5
3.0
2.5
2.0
1.5
1.0
0.5
ppm
6.84
3.44
1.60
15.92
1.81
6.84
7.87
5.71
46.96

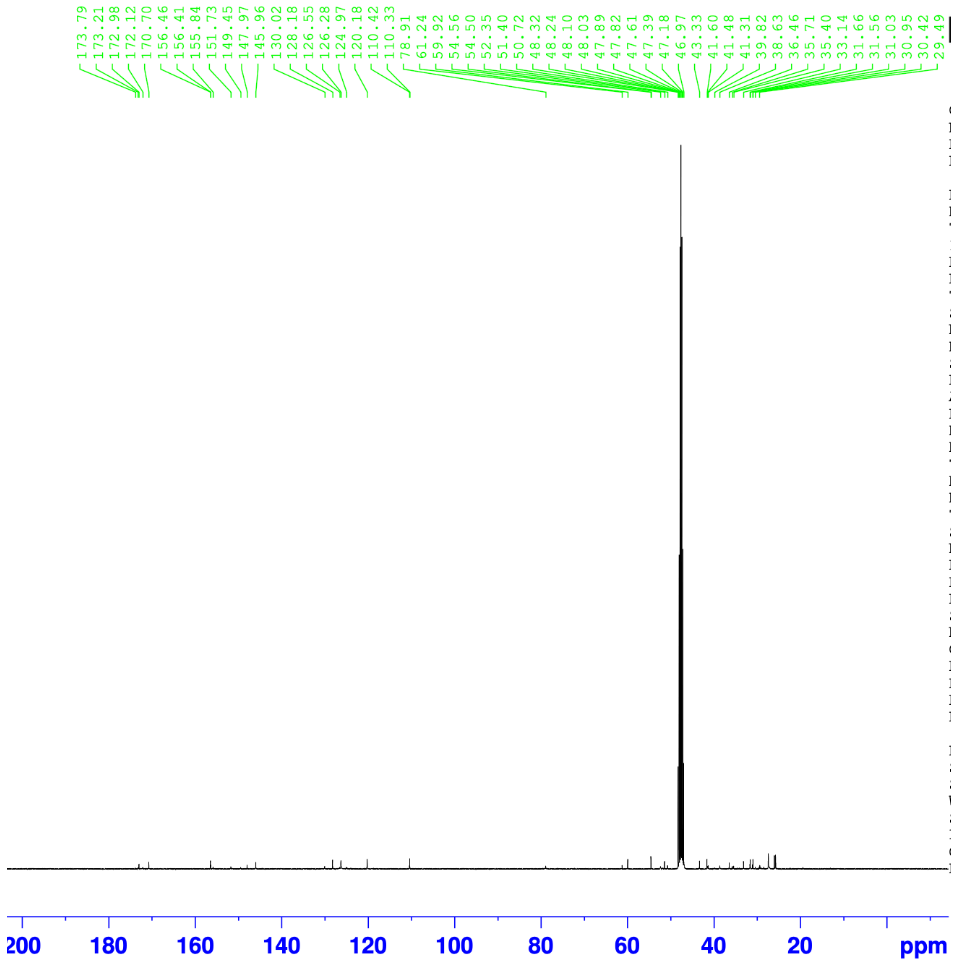
173.79
173.21
172.98
172.12
170.70
156.46
156.41
155.84
151.73
149.45
147.97
145.96
130.02
128.18
126.55
126.28
124.97
120.18
110.42
110.33
78.91
61.24
59.92
54.56
54.50
52.35
51.40
50.72
48.32
48.24
48.10
48.03
47.89
47.82
47.61
47.39
47.18
46.97
43.33
41.60
41.48
41.31
39.82
38.63
36.46
35.71
35.40
33.14
31.66
31.56
31.03
30.95
30.42
29.49
200
180
160
140
120
100
80
60
40
20
ppm

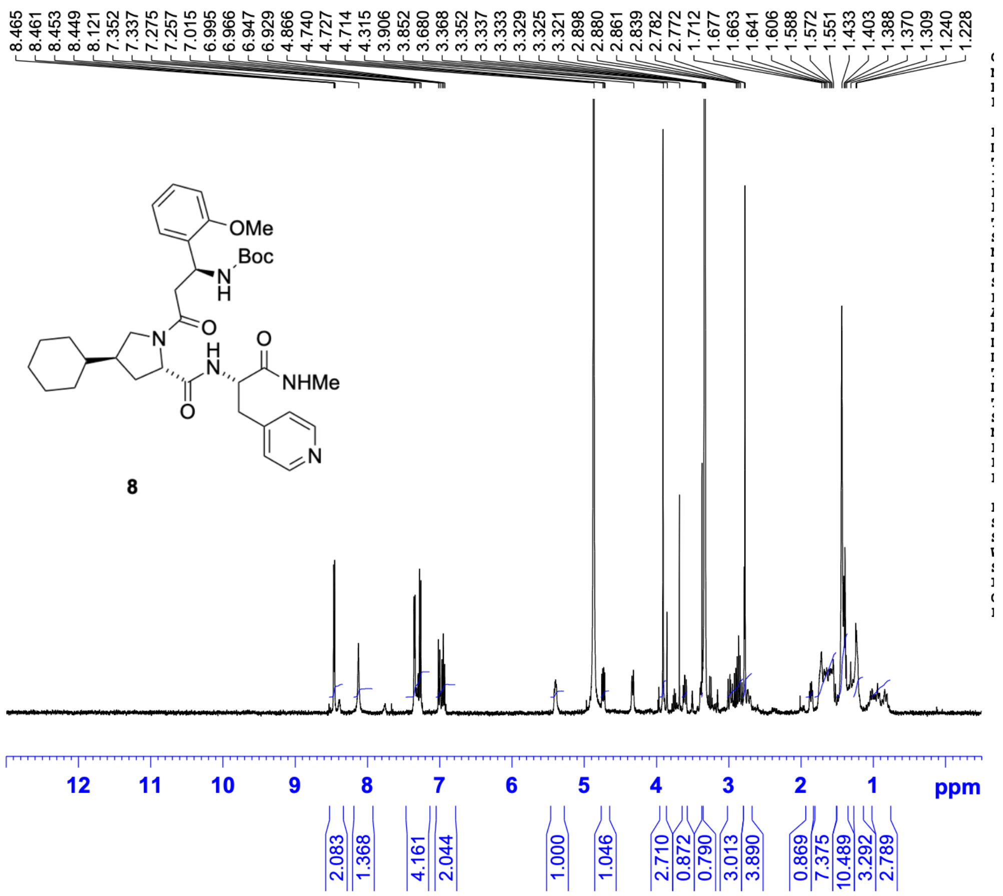
8.465 8.461 8.453 8.449 8.121 7.352 7.337 7.275 7.257 7.015 6.995 6.966 6.947 6.929 4.866 4.740 4.727 4.714 4.315 3.906 3.852 3.680 3.368 3.352 3.337 3.333 3.329 3.325 3.321 2.898 2.880 2.861 2.839 2.782 2.772 1.712 1.677 1.663 1.641 1.606 1.588 1.572 1.551 1.433 1.403 1.388 1.370 1.309 1.240 1.228
OMe
Boc
N
H
O
N
H
N
O
NHMe
O
N
8
12 11 10 9 8 7 6 5 4 3 2 1 ppm
2.083 1.368 4.161 2.044 1.000 1.046 2.710 0.872 0.790 3.013 3.890 0.869 7.375 10.489 3.292 2.789

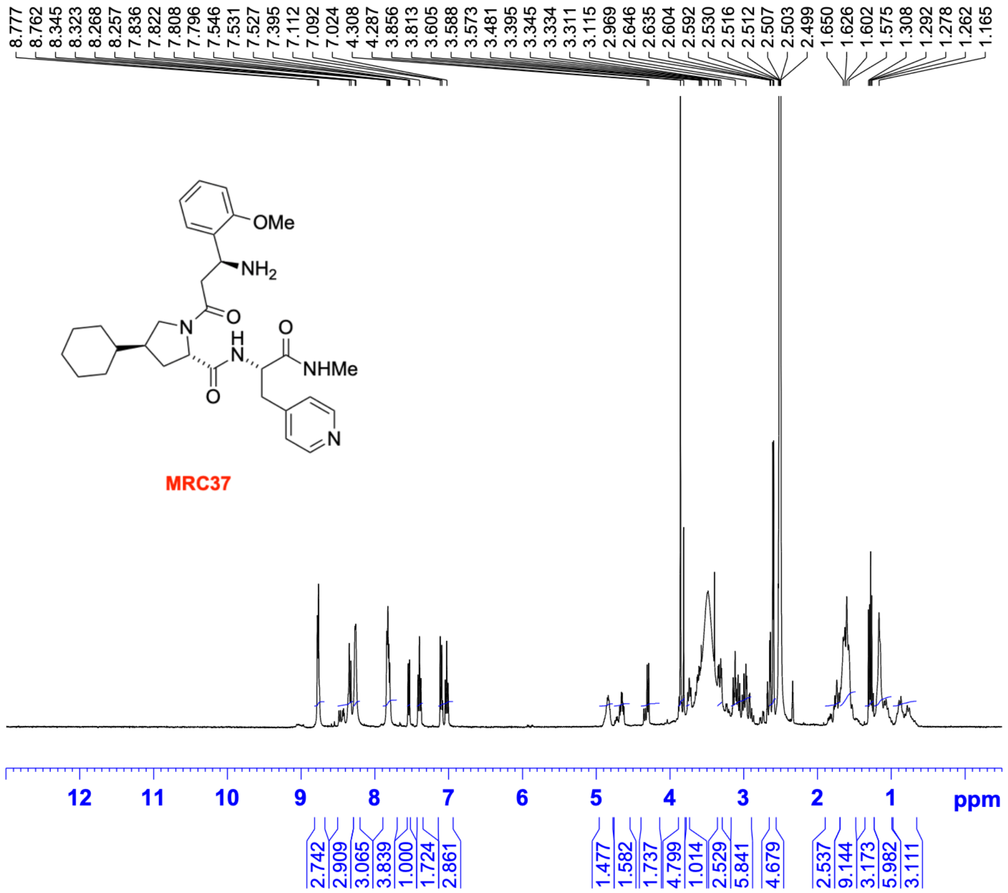
8.777 8.762 8.345 8.323 8.268 8.257 7.836 7.822 7.808 7.796 7.546 7.531 7.527 7.395 7.112 7.092 7.024 4.308 4.287 3.856 3.813 3.605 3.588 3.573 3.481 3.395 3.345 3.334 3.311 3.115 2.969 2.646 2.635 2.604 2.592 2.530 2.516 2.512 2.507 2.503 2.499 1.650 1.626 1.602 1.575 1.308 1.292 1.278 1.262 1.165
OMe
NH2
O
N
H
N
O
NHMe
O
N
MRC37
12 11 10 9 8 7 6 5 4 3 2 1 ppm
2.742 2.909 3.065 3.839 1.000 1.724 2.861 1.477 1.582 1.737 4.799 1.014 2.529 5.841 4.679 2.537 9.144 3.173 5.982 3.111

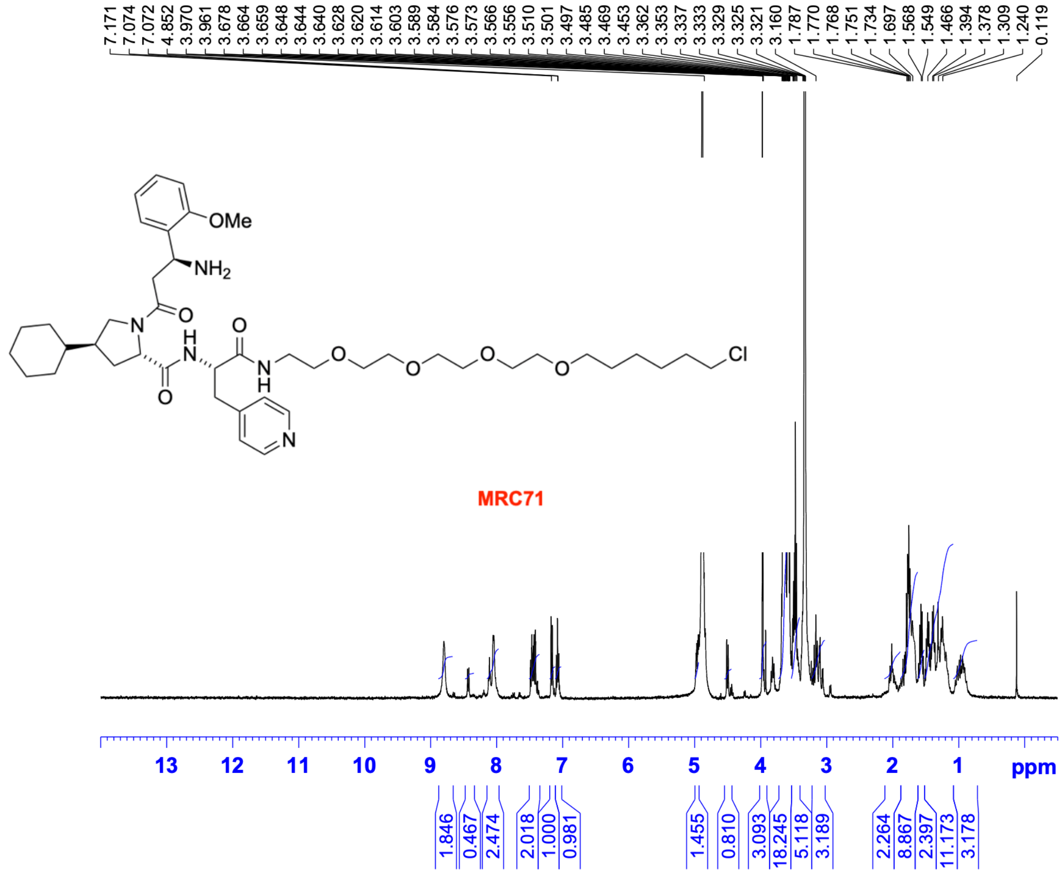
7.171
7.074
7.072
4.852
3.970
3.961
3.678
3.664
3.659
3.648
3.644
3.640
3.628
3.620
3.614
3.603
3.589
3.584
3.576
3.573
3.566
3.556
3.510
3.501
3.497
3.485
3.469
3.453
3.362
3.353
3.337
3.333
3.329
3.325
3.321
3.160
1.787
1.770
1.768
1.751
1.734
1.697
1.568
1.549
1.466
1.394
1.378
1.309
1.240
0.119
OMe
NH2
N
O
H
N
O
N
H
O
O
O
O
Cl
O
N
13
12
11
10
9
8
7
6
5
4
3
2
1
ppm
1.846
0.467
2.474
2.018
1.000
0.981
1.455
0.810
3.093
18.245
5.118
3.189
2.264
8.867
2.397
11.173
3.178

MRC71

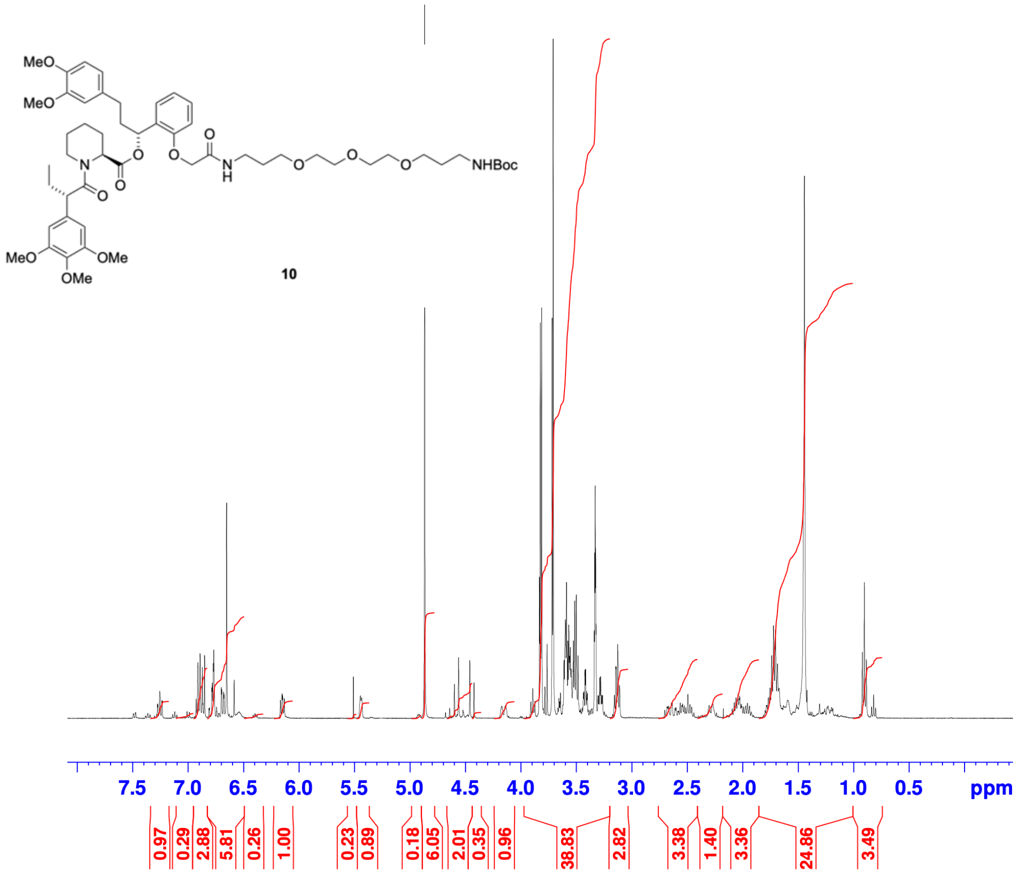
MeO
MeO
N
O
O
O
O
O
N
H
O
O
O
NHBoc
MeO
OMe
OMe
7.5
7.0
6.5
6.0
5.5
5.0
4.5
4.0
3.5
3.0
2.5
2.0
1.5
1.0
0.5
ppm
0.97
0.29
2.88
5.81
0.26
1.00
0.23
0.89
0.18
6.05
2.01
0.35
0.96
38.83
2.82
3.38
1.40
3.36
24.86
3.49

10

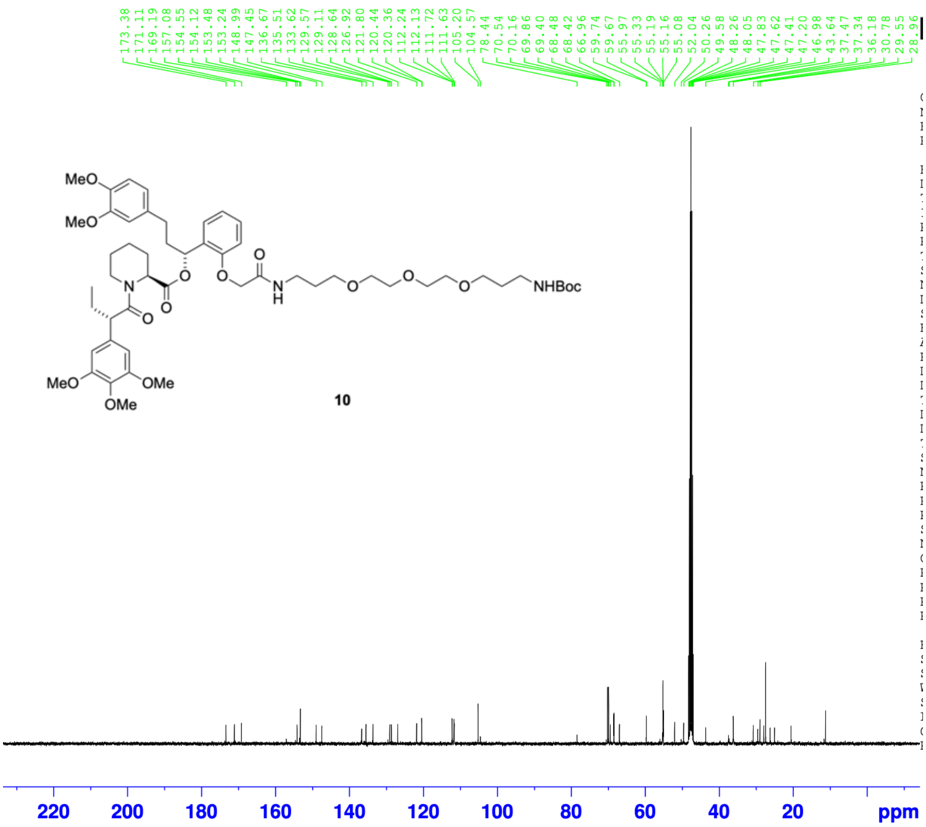
173.38
171.11
169.19
157.08
154.55
154.12
153.48
153.24
148.99
147.45
136.67
135.51
133.62
129.57
129.11
128.64
126.92
121.80
120.44
120.36
112.24
112.13
111.72
111.63
105.20
104.57
78.44
70.54
70.16
69.86
69.40
68.48
68.42
66.96
59.74
59.67
55.97
55.33
55.19
55.16
55.08
52.04
50.26
49.58
48.26
48.05
47.83
47.62
47.41
47.20
46.98
43.64
37.47
37.34
36.18
30.78
29.55
28.96
MeO
MeO
O
N
O
O
O
O
N
H
O
O
O
NHBoc
MeO
OMe
OMe
220
200
180
160
140
120
100
80
60
40
20
ppm

10

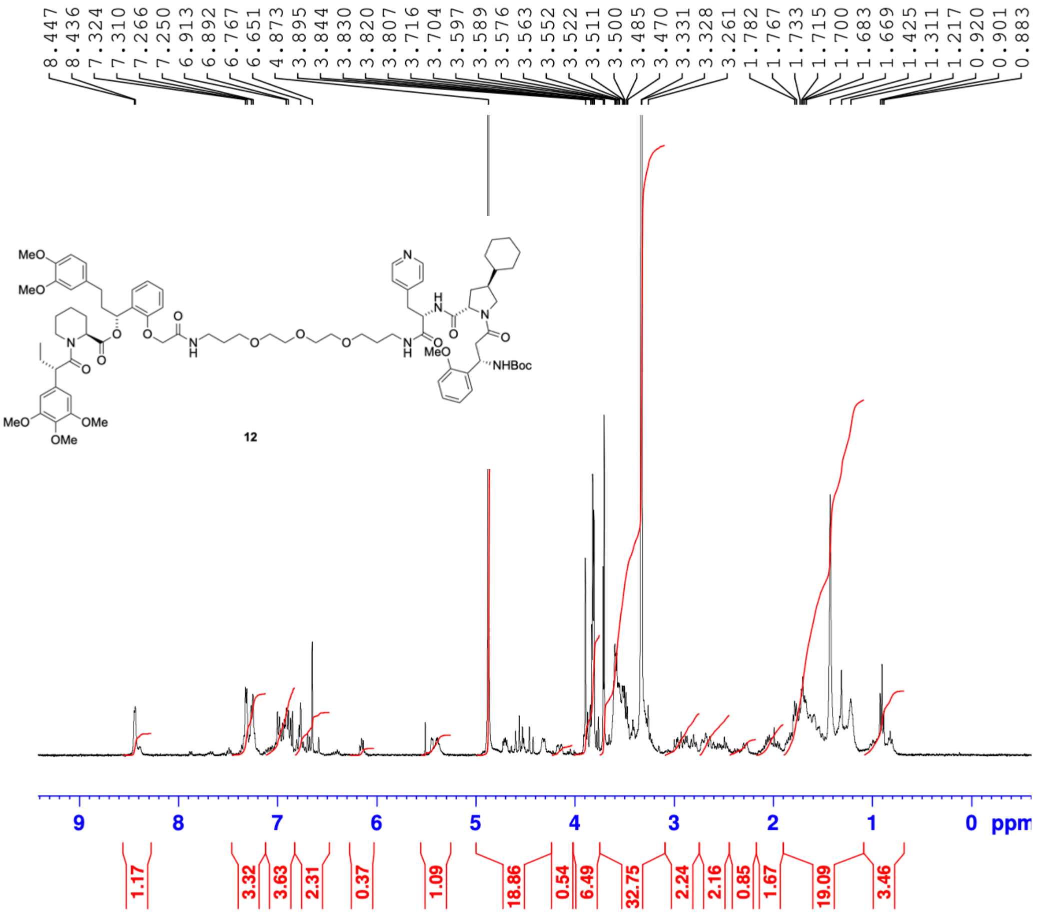
8.447
8.436
7.324
7.310
7.266
7.250
6.913
6.892
6.767
6.651
4.873
3.895
3.844
3.830
3.820
3.807
3.716
3.704
3.597
3.589
3.576
3.563
3.552
3.522
3.511
3.500
3.485
3.470
3.331
3.328
3.261
1.782
1.767
1.733
1.715
1.700
1.683
1.669
1.425
1.311
1.217
0.920
0.901
0.883
MeO
MeO
N
O
O
O
O
N
H
O
O
O
N
H
O
N
H
O
N
O
MeO
NHBoc
MeO
OMe
OMe
9
8
7
6
5
4
3
2
1
0
ppm
1.17
3.32
3.63
2.31
0.37
1.09
18.86
0.54
6.49
32.75
2.24
2.16
0.85
1.67
19.09
3.46

12

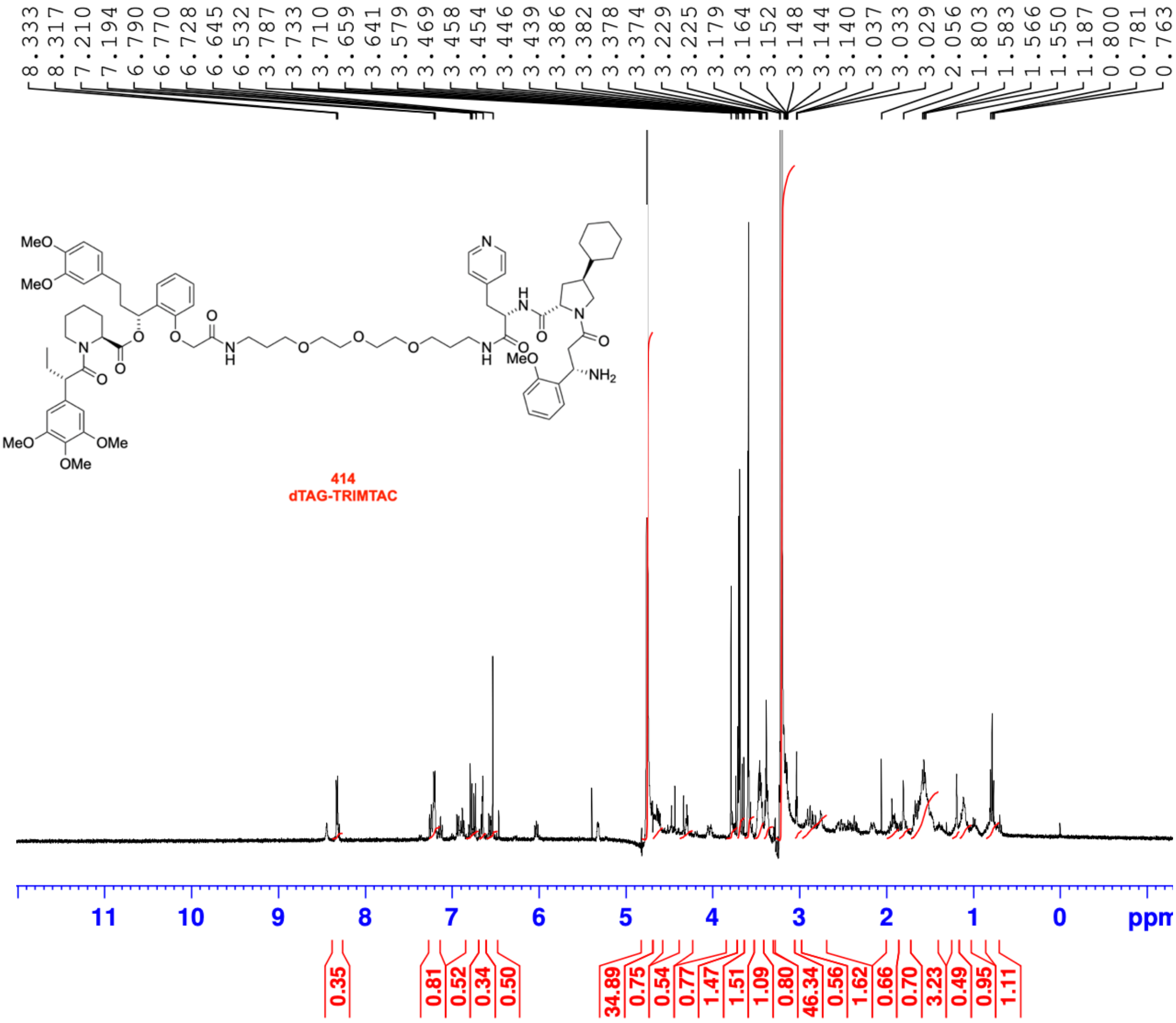

414
dTAG-TRIMTAC

Supplementary Figure Legends

Supplementary Figure 1: Identification of ligands for TRIM21 PRYSPRY. (a) SDS-PAGE gels stained with Instant blue showing that the activity of T21 PRYSPRY is maintained when covalently bound to beads; left: shows the amount of IgG that can be recovered in the experiment. Middle: Beads reacted with either no protein, control protein or T21 PRYSPRY was incubated with the IgG, washed and the bound fraction analysed. Right: Similar as the middle experiment but where Protein AG was included when beads were incubated with IgG to show that binding to TRIM21 PRYSPRY is specific. **(b)** MRC37, MRC37 and MRC38 compound structures. **(c)** Thermal stabilisation of TRIM21 PRYSPRY by indicated ligands as compared to melting temperature in the presence of solvent (DMSO) alone. Plotted difference in melting temperature (ΔT_m) as an average (± s.e.m.) of two independent replicates (n=2). **(d)** Tryptophan quenching experiment, where the compounds are titrated against a constant T21 PRYSPRY and the intrinsic fluorescence of the protein is monitored to follow binding of the compounds to the protein. Data is expressed as a percentage of fluorescence (RFU) in the absence of compound. **(e)** Fluorescence polarization displacement experiment, where T21 PRYSPRY labelled with Alexa-488 is bound to IgG Fc and titrated against the compounds to determine if they can displace Fc, as determined by the change in polarisation of the fluorescence. The full black circles show a titration of IgG Fc against 488-labelled T21 PRYSPRY. **(f)** Affinity of indicated compounds to wild-type (WT) T21 PRYSPRY and selected mutants as measured by quenching of intrinsic tryptophan fluorescence. Values are Kd in µM.

Supplementary Figure 2: haloTRIMTAC compounds. Compound structures for MRC71, MRC109 and MRC111.

Supplementary Figure 3: Kinetics of degradation of oligomeric substrate Cavin1-mEGFP-Halo. RPE1 cells expressing Cavin1-mEGFP-Halo were treated with haloTRIMTAC MRC71 and the fluorescence monitored using live microscopy. An exemplar cell showing that the oligomeric membrane associated protein is rapidly degraded but diffuse cytoplasmic protein remains. Scale bar = 10 µm

Supplementary Figure 4: dTAG-TRIMTAC rapidly and efficiently degrades Cavin1-mEGFP-FKBP(F36V). (a) Two different FKBP(F36V)-tagged targets for TRIMTACs; mEGFP-FKBP(F36V) is a monomeric protein, Cavin1-mEGFP-FKBP(F36V) forms oligomers within caveoli membrane structures. **(b)** Representative images of RPE-1 stable cell lines expressing the difference protein constructs; mEGFP-FKBP(F36V) exhibits diffuse cytosolic localisation, Cavin1-mEGFP-FKBP(F36V) exhibits bright signal at the cell membrane indicative of caveoli structures. Yellow dotted line shows the cell outline. **(c-d)** MRC414 compounds selectively degrade oligomeric Cavin1-mEGFP-FKBP(F36V) over monomeric mEGFP-FKBP(F36V) and degradation is TRIM21 dependent (d). Graphs show the integrated density of GFP fluorescence (in relative fluorescence units, RFU) normalized to total cell area (phase) from 1×10^4 cells and expressed as a fraction of the DMSO control. Data is expressed as mean and s.e.m. from *n* = 4 technical replicates. Representative examples from *n* = 2 independent experiments.

Supplementary Figure 5: dTAG-TRIMTAC degrades RIPK3 oligomers as they assemble. Live imaging of mEGFP-FKBP(F36V)-RIPK3 shows that MRC414 degrades RIPK3 oligomers as they assemble whereas GSK'872 does not affect RIPK3 oligomerization. Scale bar = 20 µm.

Supplementary Figure 6: FKBP(F36V)-tau assemblies induce accumulation of intracellular pS422-positive aggregates. Neural cultures were treated with indicated volumes of FKBP(F36V)-tau assemblies for 7 days, followed by methanol fixation and staining with the MAP2 and pS422 (anti-phospho-tau) antibodies. Counts of pS422 puncta were normalized against MAP2 neuronal coverage per image. N = 3 biological replicates.

Supplementary Table 1: Data collection and refinement statistics. Information for x-ray structure determination.

Supplementary Data 1: List of materials. Plasmids, sequences and proteins.

Supplementary Methods: Chemical synthesis. Detailed synthetic description for all compounds.
