# Supplementary material for: State-selective small molecule degraders that preferentially remove aggregates and oligomers
Source: Nat Commun. 2025 Nov 25;16:10486. doi: 10.1038/s41467-025-65454-z (PMC12647892; doi:10.1038/s41467-025-65454-z)
Supplement: Supplementary file 3 — Reporting Summary [file 41467_2025_65454_MOESM3_ESM.pdf]

nature portfolio

Corresponding author(s): James LC

Last updated by author(s): 2025/27/06

# Reporting Summary

Nature Portfolio wishes to improve the reproducibility of the work that we publish. This form provides structure for consistency and transparency in reporting. For further information on Nature Portfolio policies, see our Editorial Policies and the Editorial Policy Checklist.

## Statistics

For all statistical analyses, confirm that the following items are present in the figure legend, table legend, main text, or Methods section.

| n/a | Confirmed | |
|---|---|---|
| ☐ | ☒ | The exact sample size (*n*) for each experimental group/condition, given as a discrete number and unit of measurement |
| ☐ | ☒ | A statement on whether measurements were taken from distinct samples or whether the same sample was measured repeatedly |
| ☐ | ☒ | The statistical test(s) used AND whether they are one- or two-sided<br>*Only common tests should be described solely by name; describe more complex techniques in the Methods section.* |
| ☒ | ☐ | A description of all covariates tested |
| ☐ | ☒ | A description of any assumptions or corrections, such as tests of normality and adjustment for multiple comparisons |
| ☐ | ☒ | A full description of the statistical parameters including central tendency (e.g. means) or other basic estimates (e.g. regression coefficient) AND variation (e.g. standard deviation) or associated estimates of uncertainty (e.g. confidence intervals) |
| ☐ | ☒ | For null hypothesis testing, the test statistic (e.g. $F$, $t$, $r$) with confidence intervals, effect sizes, degrees of freedom and $P$ value noted<br>*Give P values as exact values whenever suitable.* |
| ☒ | ☐ | For Bayesian analysis, information on the choice of priors and Markov chain Monte Carlo settings |
| ☒ | ☐ | For hierarchical and complex designs, identification of the appropriate level for tests and full reporting of outcomes |
| ☒ | ☐ | Estimates of effect sizes (e.g. Cohen's $d$, Pearson's $r$), indicating how they were calculated |

*Our web collection on statistics for biologists contains articles on many of the points above.*

## Software and code

Policy information about availability of computer code

| | |
|---|---|
| Data collection | CCP4 Package (Winn, M. D. et al. Overview of the CCP4 suite and current developments. Acta Crystallogr D Biol Crystallogr 67, 235-242, doi:10.1107/S0907444910045749 (2011).) |
| Data analysis | Prism Version 9<br>IncuCyte Software, Sartorius, version 2025A<br>Compass software for simple western, Bio-Techne, version 7.0<br>PyMOL version 3.1<br>ImageJ/FIJI version 2.9.0 |

For manuscripts utilizing custom algorithms or software that are central to the research but not yet described in published literature, software must be made available to editors and reviewers. We strongly encourage code deposition in a community repository (e.g. GitHub). See the Nature Portfolio guidelines for submitting code & software for further information.

## Data

Policy information about availability of data

All manuscripts must include a data availability statement. This statement should provide the following information, where applicable:

- Accession codes, unique identifiers, or web links for publicly available datasets
- A description of any restrictions on data availability
- For clinical datasets or third party data, please ensure that the statement adheres to our policy

All data in the manuscript is included in the accompanying Source Data files with the exception of x-ray structures that have been deposited with the PDB codes 9Q9O, 9Q9P, 9Q9Q, 9Q9R,and 9R40

## Research involving human participants, their data, or biological material

Policy information about studies with human participants or human data. See also policy information about sex, gender (identity/presentation), and sexual orientation and race, ethnicity and racism.

| | |
|---|---|
| Reporting on sex and gender | Not Applicable |
| Reporting on race, ethnicity, or other socially relevant groupings | Not Applicable |
| Population characteristics | Not Applicable |
| Recruitment | Not Applicable |
| Ethics oversight | Not Applicable |

Note that full information on the approval of the study protocol must also be provided in the manuscript.

# Field-specific reporting

Please select the one below that is the best fit for your research. If you are not sure, read the appropriate sections before making your selection.

[x] Life sciences [ ] Behavioural & social sciences [ ] Ecological, evolutionary & environmental sciences

For a reference copy of the document with all sections, see nature.com/documents/nr-reporting-summary-flat.pdf

# Life sciences study design

All studies must disclose on these points even when the disclosure is negative.

| | |
|---|---|
| Sample size | No statistical method was used to predetermine the sample size. |
| Data exclusions | There were no data exclusions. |
| Replication | No in vivo experiments were performed. For in vitro experiments, data was collected with technical replicates and experiments were repeated to obtain biological replicates and all replicates were successful. |
| Randomization | Not applicable |
| Blinding | Not applicable |

# Reporting for specific materials, systems and methods

We require information from authors about some types of materials, experimental systems and methods used in many studies. Here, indicate whether each material, system or method listed is relevant to your study. If you are not sure if a list item applies to your research, read the appropriate section before selecting a response.

## Materials & experimental systems

| n/a | Involved in the study |
|---|---|
| | ☒ Antibodies |
| | ☒ Eukaryotic cell lines |
| ☒ | ☐ Palaeontology and archaeology |
| | ☒ Animals and other organisms |
| ☒ | ☐ Clinical data |
| ☒ | ☐ Dual use research of concern |
| ☒ | ☐ Plants |

## Methods

| n/a | Involved in the study |
|---|---|
| ☒ | ☐ ChIP-seq |
| ☒ | ☐ Flow cytometry |
| ☒ | ☐ MRI-based neuroimaging |

# Antibodies

Antibodies used

humanised anti-hexon IgG1 9C12. Dilution is variable and given in the relevant figures
mouse anti-actin. Capillary-based immunoblotting 1:100
anti-mouse NIR, Capillary-based immunoblotting secondary antibody 1:1000
rabbit anti-GFP, Capillary-based immunoblotting 1:10,000
anti-rabbit HRP, Capillary-based immunoblotting secondary antibody 1:1000

Validation

Validation details are provided in the following websites:

https://doi.org/10.4049/jimmunol.1502601
bio-techne; MAB8929
bio-techne; 043-821
novus; NB600-303
bio-techne; 042-206

# Eukaryotic cell lines

Policy information about cell lines and Sex and Gender in Research

Cell line source(s)

See Supplementary Data 1:

HEK293T ATCC; CRL-3216
U2OS ATCC; HTB-96
hTERT-RPE-1 ATCC; CRL-4000
hTERT-RPE-1 H2B-mEGFP This paper
hTERT-RPE-1 TRIM21 KO https://doi.org/10.1038/s41594-021-00560-2
hTERT-RPE-1 CAV1-mEGFP-Halo This paper
hTERT-RPE-1 Cavin-1-mEGFP-Halo This paper
hTERT-RPE-1 mEGFP-Halo This paper
hTERT-RPE-1 TRIM21 KO CAV1-mEGFP-Halo This paper
hTERT-RPE-1 TRIM21 KO Cavin-1-mEGFP-Halo This paper
hTERT-RPE-1 Cavin-1-mEGFP-FKBP(F36V) This paper
hTERT-RPE-1 mEGFP-FKBP(F36V) This paper
hTERT-RPE-1 TRIM21 KO Cavin-1-mEGFP-FKBP(F36V) This paper
hTERT-RPE-1 TRIM21 KO CAV1-mEGFP-Halo This paper
hTERT-RPE-1 TRIM21 KO H2B-mEGFP mCherry-TRIM21 This paper
hTERT-RPE-1 TRIM21 KO H2B-mEGFP mCherry-TRIM21ΔPRYSPRY This paper
hTERT-RPE-1 TRIM21 KO H2B-mEGFP mCherry-TRIM21ΔRING-Box This paper
hTERT-RPE-1 FKBP(F36V)-mEGFP-RIPK3 This paper
U2OS 3xFlag-Myd88-GyrB-mEGFP-FKBP(F36V) This paper
HEK293T 0N4R P301S Tau-Venus https://doi.org/10.1073/pnas.1607215114

Authentication

Parental HEK293T, U2OS and RPE-1 cells were authenticated by the vendor. Other cells were not authenticated.

Mycoplasma contamination

All cells are routinely mycoplasma tested and are negative.

Commonly misidentified lines
(See ICLAC register)

n.a

# Animals and other research organisms

Policy information about studies involving animals; ARRIVE guidelines recommended for reporting animal research, and Sex and Gender in Research

| | |
|---|---|
| Laboratory animals | Mice C57BL/6 |
| Wild animals | none |
| Reporting on sex | An equal number of male and female mice were used of P0 and P1 for ex vivo experiments and pooled for primary neuron cultures. |
| Field-collected samples | none |
| Ethics oversight | All animal work was licensed under the UK Animals (Scientific Procedures) Act 1986 and approved by the Medical Research Council Animal Welfare and Ethical Review Body. |

Note that full information on the approval of the study protocol must also be provided in the manuscript.

# Plants

| | |
|---|---|
| Seed stocks | Not applicable |
| Novel plant genotypes | Not applicable |
| Authentication | Not applicable |
